# Diffusional Electron Transport Coupled to Thermodynamically Driven Electron Transfers in Redox-Conductive Multivariate Metal–Organic Frameworks

**DOI:** 10.1021/jacs.4c01401

**Published:** 2024-04-19

**Authors:** Jingguo Li, Amol Kumar, Sascha Ott

**Affiliations:** † Department of Chemistry—Ångström Laboratory, Uppsala University, Box 523, 75120 Uppsala, Sweden; ‡ Wallenberg Initiative Materials Science for Sustainability, Department of Chemistry—Ångström Laboratory, Uppsala University, Box 523, 75120 Uppsala, Sweden

## Abstract

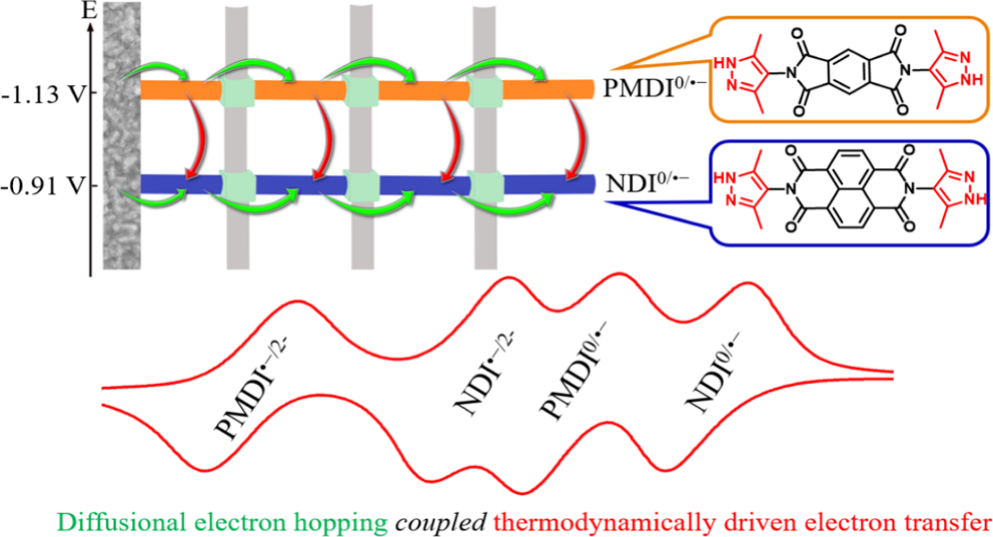

The development of redox-conductive metal–organic frameworks (MOFs) and the fundamental understanding of charge propagation through these materials are central to their applications in energy storage, electronics, and catalysis. To answer some unresolved questions about diffusional electron hopping transport and redox conductivity, mixed-linker MOFs were constructed from two statistically distributed redox-active linkers, pyromellitic diimide bis-pyrazolate (PMDI) and naphthalene diimide bis-pyrazolate (NDI), and grown as crystalline thin films on conductive fluorine-doped tin oxide (FTO). Owing to the distinct redox properties of the linkers, four well-separated and reversible redox events are resolved by cyclic voltammetry, and the mixed-linker MOFs can exist in five discrete redox states. Each state is characterized by a unique spectroscopic signature, and the interconversions between the states can be followed spectroscopically under operando conditions. With the help of pulsed step-potential spectrochronoamperometry, two modes of electron propagation through the mixed-linker MOF are identified: diffusional electron hopping transport between linkers of the same type and a second channel that arises from thermodynamically driven electron transfers between linkers of different types. Corresponding to the four redox events of the mixed-linker MOFs, four distinct bell-shaped redox conductivity profiles are observed at a steady state. The magnitude of the maximum redox conductivity is evidenced to be dependent on the distance between redox hopping sites, analogous to the situation for apparent electron diffusion coefficients, *D*_e_^app^, that are obtained in transient experiments. The design of mixed-linker redox-conductive MOFs and detailed studies of their charge transport properties present new opportunities for future applications of MOFs, in particular, within electrocatalysis.

## Introduction

Electroactive metal–organic frameworks (MOFs) are fascinating research targets due to their broad spectrum of potential applications, including electrocatalysis, energy storage, electrochemical sensors, reconfigurable electronics, etc.^1−4^ Meanwhile, their well-defined structures and high modularity also offer opportunities for fundamental studies of electron transport in porous materials and for establishing composition–property relationships.^5,6^ In general, there are two fundamentally different strategies to engineer electric conductivity into conventionally dielectric MOFs, either through band-like electron transport or through electron hopping.^1^ The former is essentially rooted in the band theory of solid-state physics,^7^ and in the context of MOFs, such delocalized bands often result from either metal-linker d–π conjugation or linker-based π–π stacking.^8−10^ Depending on the degree of band dispersion, such MOFs can exhibit semiconductor-like or even metal-like electric conductivities.^11−15^ In contrast to MOFs with band conduction, electron hopping transport operates between electronically isolated molecular sites of different oxidation states,^6,16,17^ with the resulting redox conductivity of the MOF being fundamentally determined by its redox state.^18^

Among the redox-conductive MOFs, Zn(NDI) (NDI = naphthalene diimide bis-pyrazolate) has been extensively studied, mainly in the context of electrochromism and fundamental redox hopping behaviors.^19−21^ This MOF consists of infinite chains of Zn^2+^ ions as redox-inert secondary building units and redox-active NDI linkers, which are electronically isolated. Consequently, electrons propagate through the MOF by hopping between neighboring NDI linkers, a process that is coupled to the diffusion migration of charge balancing counterions.^22^ Recently, we demonstrated on Zn(NDI)@FTO thin films that redox hopping is a fundamental prerequisite of redox conductivity.^18^ Redox conductivity maximizes when the reduced and oxidized redox-active “hopping sites” are in a 50:50 ratio, which, according to the Nernst equation, is achieved at an applied potential that is identical to the midpoint potential of the NDI linker. This characteristic potential-dependent conductivity feature is a unique asset for designing functional MOF materials.^16,23−25^ While the field’s understanding of redox conducting MOFs has increased dramatically in recent years, there are still various open questions, some of which we address in the multivariant redox-active MOFs herein for the first time.^26^

A unique mixed-linker Zn(NDI)_*x*_(PMDI)_*y*_ (PMDI = pyromellitic diimide bis-pyrazolate) MOF is presented that consists of two similarly sized linkers but different electrochemical and electronic properties. The ratio of the two linkers can be varied from 0 to 100% during the solvothermal synthesis, and thin films of good crystallinity can be grown on fluorine-doped tin oxide (FTO) substrates. The difference in electron affinity between PMDI and NDI sets the foundation for designing electroactive MOFs with intriguing electrochemical, optoelectronic, and redox conductivity properties. In particular, the well-separated spectroscopic signatures of the associated electronic states provide an excellent platform to probe electron-transfer processes between linkers of different redox potentials.^27^ Moreover, the mixed-linker MOF allows for fundamental studies of the redox hopping process under transient and steady-state conditions. In particular, it will be shown that the steady-state redox conductivity shows a dependency on the hopping distance, similar to the situation for apparent electron diffusion coefficients *D*_e_^app^ that are determined under transient conditions.^28−30^ The present study thus bridges our mechanistic understandings of redox hopping across different experiments, which is important for the design and application of redox-active MOFs.

## Results and Discussion

### Linker Design, Mixed-Linker MOF Synthesis, and Basic Characterizations

**Figure 1:**
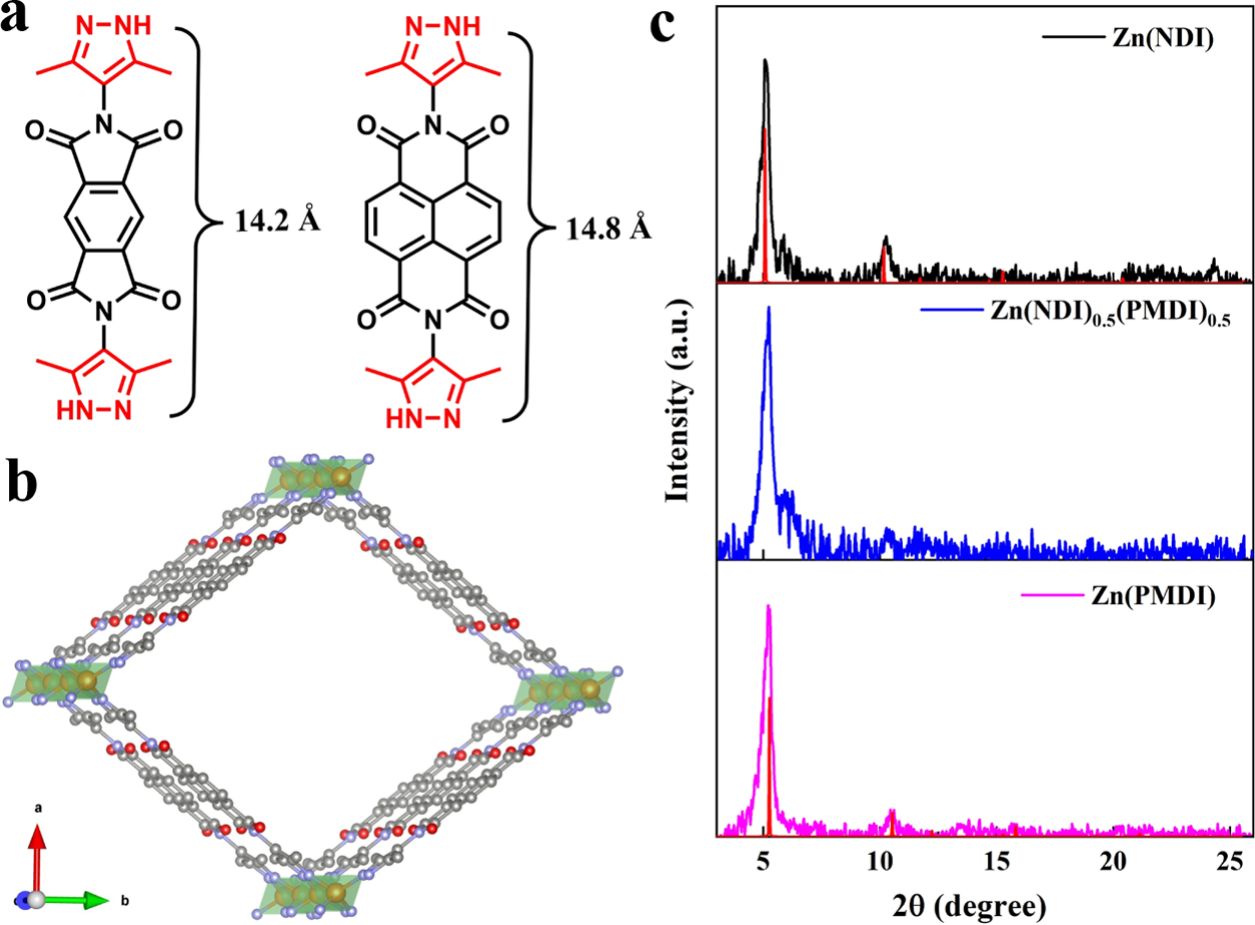
Chemical structure of the two bis-pyrazole-terminated diimide linkers, PMDI and NDI, with comparable head-to-head distances of 14.2 and 14.8 Å, respectively (a). The crystal structure of Zn(NDI) viewing along the c direction where pyrazolate head groups are bridged by an infinite chain of tetrahedral Zn^2+^ ions and hydrogen atoms are omitted for clarity; C, N, O, and Zn atoms or ions are presented with gray, blue, red, and brown spheres, respectively (b). Experimental thin film X-ray diffraction (XRD) data of monolinker Zn(NDI), Zn(PMDI), and mixed-linker Zn(NDI)_0.5_(PMDI)_0.5_ on fluorine-doped tin oxide surface (FTO) together with simulated powder XRD data (c).

To construct MOFs with two electrochemically distinct linkers, bis-pyrazole-terminated diimides of PMDI and NDI (PMDI = pyromellitic diimide bis-pyrazoles, NDI = naphthalene diimide bis-pyrazoles; Figure 1a) with comparable length and molecular topology were synthesized (see the Supporting Information (SI) for more details of both linkers, ^1^H NMR data in Figures S1 and S2, absorption data in Figures S3 and S4, and cyclic voltammetry data in Figures S5 and S6).^21^ Their equivalent molecular size and linkage chemistry are important for making mixed-linker MOFs with varying linker stoichiometry and should also result in the statistical distribution of the linkers within the materials.^31^ To facilitate the fundamental redox hopping studies, high-quality mixed-linker MOF thin films as well as monolinker reference electrodes were prepared on conductive FTO substrates by varying the feeding ratio of NDI/PMDI linkers (100:0, 80:20, 50:50, 20:80, and 0:100) during the solvothermal synthesis (see Experimental Section for details).^19,21^ The resulting linker ratio in the mixed-linker MOFs was determined by integration of the corresponding redox waves in slow scan rate cyclic voltammetry (CV) experiments (Figures S10–S12, with more discussions below). As envisaged, an excellent correlation between the feeding ratio of the linkers and their incorporation in the MOF is observed (Figure S13), and the chemical composition of the mixed-linker MOF is thus well-controlled in a bottom-up manner. The correlation between the feeding ratio and linker stoichiometry also points toward the absence of any homolinker domains in the bulk film, and that the mixed-linker MOFs have a statistical linker distribution. This is particularly important for designing electroactive MOFs with tunable redox hopping characteristics. The surface morphology and thickness of the FTO-grown thin films were examined by scanning electron microscopy (SEM), each displaying homogeneous and compact top surfaces (Figure S7) with film thicknesses of 500–800 nm according to cross-sectional images (Figure S8). In general, the microcrystallites are getting smaller and the surface morphology becomes rougher as the proportion of PMDI linker increases. Nevertheless, all MOF thin films show good crystallinity, as indicated by thin film X-ray diffraction (XRD) characterizations (Figure 1c). Two prominent diffraction peaks, corresponding to (110) and (220) planes, are consistently observed and shift slightly to lower Bragg angles when going from the Zn(PMDI) film to Zn(NDI) film (for a more comprehensive comparison, see Figure S9). Comparison with the simulated structural models indicates a preferred orientation of the MOF crystallites in these thin films, where the *c*-axis is parallel to the conductive FTO surface (the structural model in Figure 1b was built in analogy to Dincă and co-workers’ report^32^). The MOFs adopt a common monoclinic crystal structure that is identical to that of the monolinker counterparts (Figure 1b) where pyrazolate head groups are bridged by the infinite chain of tetrahedral Zn^2+^ ions.^33,34^

**Figure 2:**
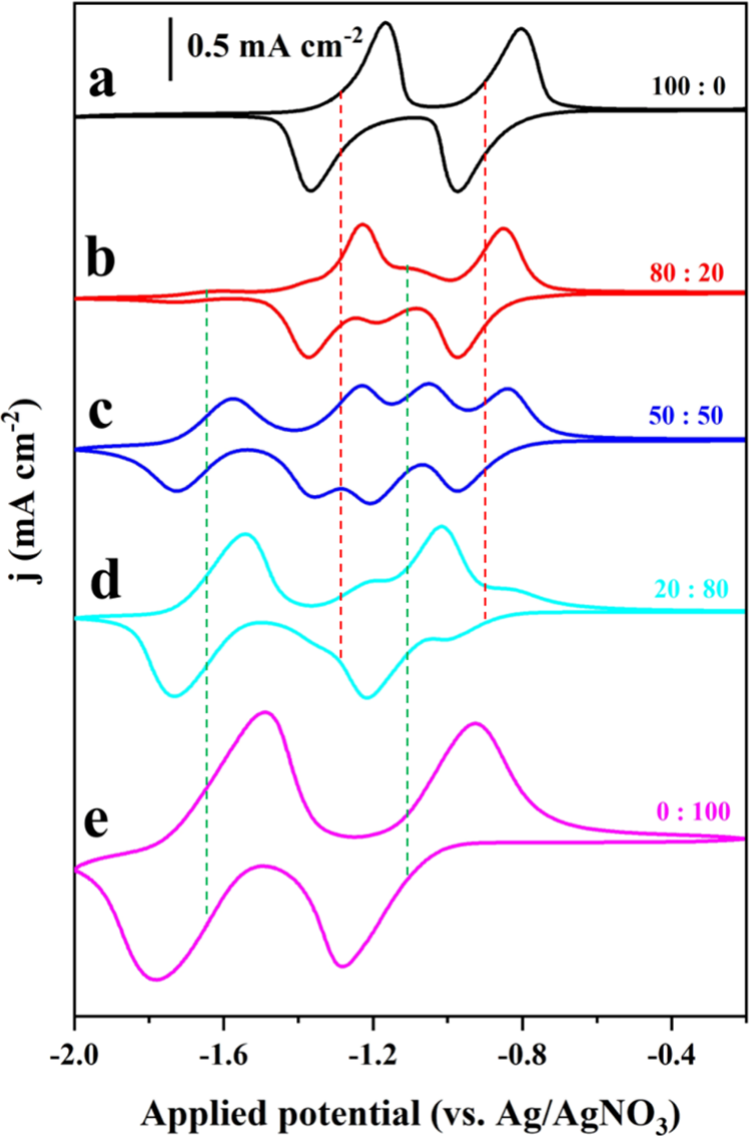
Representative thin film CV of monolinker Zn(NDI) (a), Zn(PMDI) (e), and mixed-linker Zn(NDI)_0.8_(PMDI)_0.2_ (b), Zn(NDI)_0.5_(PMDI)_0.5_ (c), and Zn(NDI)_0.2_(PMDI)_0.8_ (d) on FTO surfaces at a scan rate of 50 mV s^–1^. The ratio of NDI to PMDI in the mixed-linker MOFs was determined by the integration of respective redox waves in slow scan rate CV experiments (10 mV s^–1^, see Figures S10–S12). Half-wave potentials of linker-based NDI/NDI^•–^ and NDI^•–^/NDI^2–^ redox couples and PMDI/PMDI^•–^ and PMDI^•–^/PMDI^2–^ redox couples are labeled with red and green dashed lines, respectively. All electrochemistry data were collected in Ar-saturated DMF with KPF_6_ as the supporting electrolyte (0.1 M).

The electrochemical properties of the mixed-linker MOFs were assessed by cyclic voltammetry (CV) in a conventional three-electrode setup (more details in Experimental Section), employing the MOF@FTO as the working electrode. Very interestingly, the 50/50 mixed-linker MOF exhibits four distinct and reversible one-electron redox events with half-wave potentials at −0.91, −1.13, −1.29, and −1.65 V vs Ag/AgNO_3_ (Figure 2c). As the NDI-to-PMDI ratio increases to 80/20, the peak currents of the second and fourth redox events drop dramatically, while those of the first and third redox events remain prominent (Figure 2b). Conversely, the first and third redox events decrease and the second and fourth redox events increase in Zn(NDI)_0.2_(PMDI)_0.8_ (Figure 2d). The four waves in the mixed-linker MOFs are unambiguously assigned by comparison to homolinker reference MOFs, which show that the first and third redox events arise from the linker-based NDI/NDI^•–^ and NDI^•–^/NDI^2–^ redox couples (Figure 2a), while the second and fourth redox events originate from PMDI/PMDI^•–^ and PMDI^•–^/PMDI^2–^ redox couples (Figure 2e). The well-behaved electrochemistry data are another indication that both PMDI and NDI are statistically distributed in the mixed-linker MOFs. All four redox events in the mixed-linker MOFs are voltammetrically well separated, offering the possibility to produce five distinct redox states of the mixed-linker MOFs simply by varying the applied potential.^26^

### Spectroelectrochemistry

**Figure 3:**
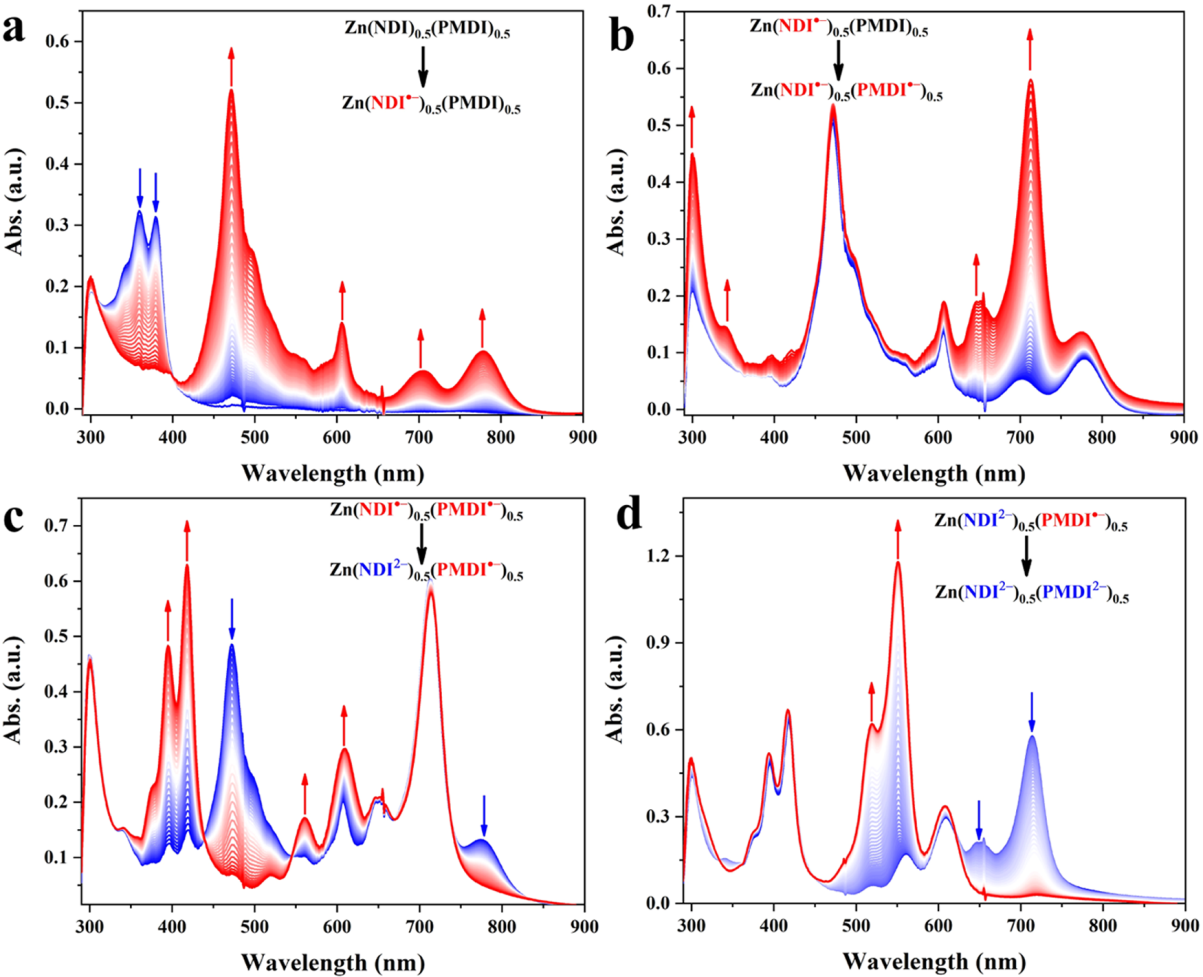
UV–vis spectroelectrochemistry measurements of a Zn(NDI)_0.5_(PMDI)_0.5_ thin film while slowly reducing it with a cathodic CV scan (10 mV s^–1^) to access the electronic signature of the different redox states: singly reduced monoradical state Zn(NDI^•–^)_0.5_(PMDI)_0.5_ (a), doubly reduced mixed radical state Zn(NDI^•–^)_0.5_(PMDI^•–^)_0.5_ (b), triply reduced mixed radical-dianion state Zn(NDI^2–^)_0.5_(PMDI^•–^)_0.5_ (c), and 4-fold reduced mixed dianion state Zn(NDI^2–^)_0.5_(PMDI^2–^)_0.5_ (d), sorted according to their appearance in the cathodic CV experiments.

**Figure 4:**
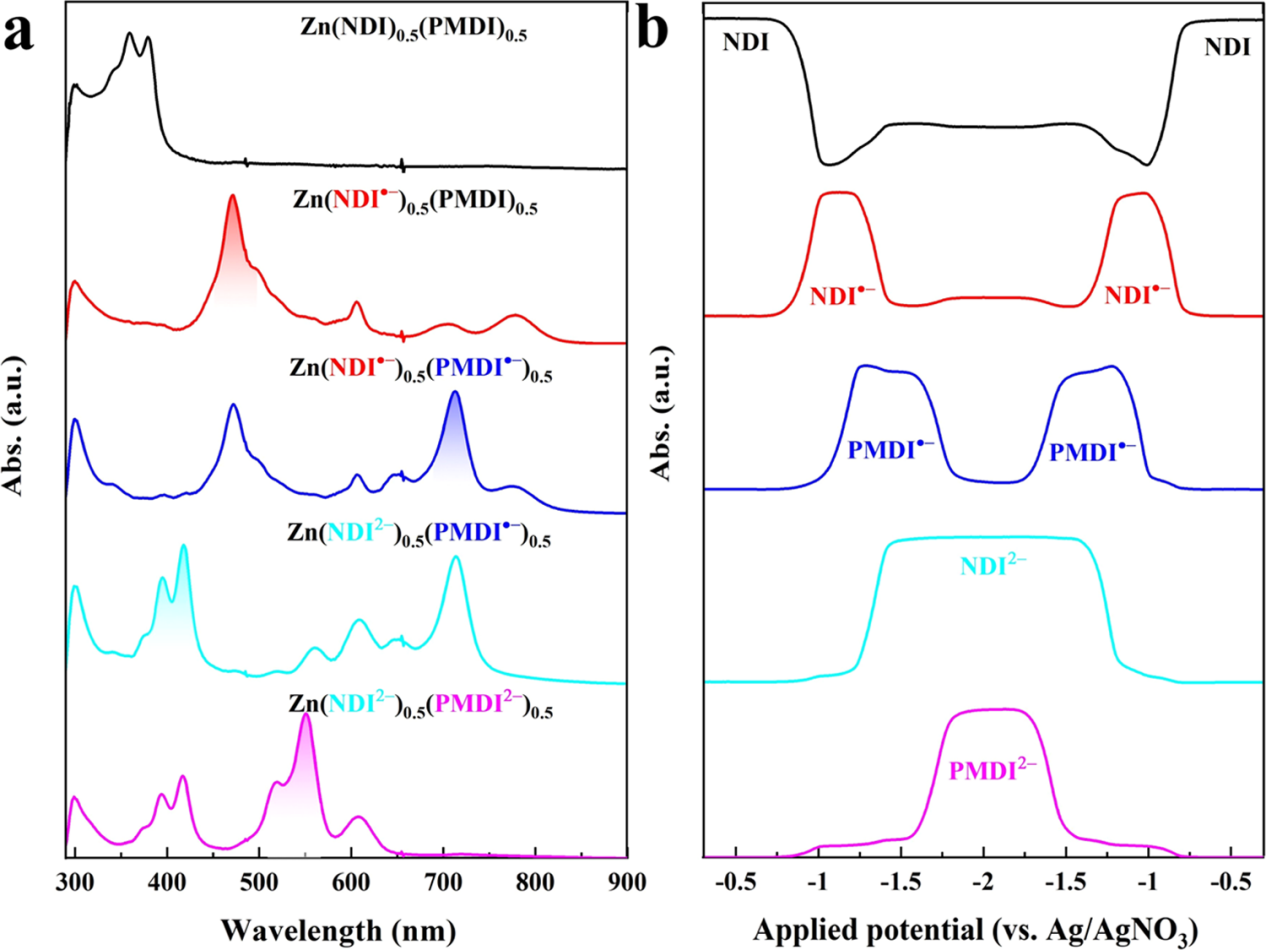
(a) Spectroscopic signatures of the Zn(NDI)_0.5_(PMDI)_0.5_ thin films in its five distinct electronic states; The unique absorption wavelength of each state is highlighted, which allows monitoring their build-up and decay. (b) Evolution of the states as a function of applied potential, following characteristic absorptions at 360 nm (NDI), 471 nm (NDI^•–^), 713 nm (PMDI^•–^), 418 nm (NDI^2–^), and 551 nm (PMDI^2–^).

To access the electronic signatures of the mixed-linker MOFs in their different redox states, operando ultraviolet–visible (UV–vis) spectroelectrochemical measurements were conducted while running a slow scan rate CV (10 mV s^–1^; experimental details in the Experimental Section). For ease of discussion, experiment data on Zn(NDI)_0.5_(PMDI)_0.5_ is presented. In its neutral state, two prominent absorption bands centered at 360 and 379 nm are observed (Figure 3a), which originate from π–π* excitations of the NDI core (assignment is assisted by absorption spectra of free NDI linkers in Figure S3, and neutral Zn(NDI) thin film in Figure S14).^35^ The PMDI linker with the smaller π-system has a larger highest occupied molecular orbital–lowest unoccupied molecular orbital (HOMO–LUMO) gap and does not feature an absorption in the visible (free PMDI linker absorbs only in the deep UV region, ∼270 nm; see Figure S4). As the cathodic scan proceeds, absorption bands of the neutral NDI linker disappear and a new set of absorption bands are assigned to the NDI^•–^ at 471, 606, 702, and 779 nm (Figure 3a). The stoichiometric transformation from neutral NDI to the one-electron reduced NDI^•–^ is evident from the isosbestic point at ∼400 nm, indicating that no intermediate species are involved on the time scale of the experiment. After the complete one-electron reduction of NDI linkers, the characteristic absorption band of PMDI^•–^ at 713 nm starts to increase (Figure 3b) as the applied potential steps into the reduction region of the PMDI linker. As the applied potential further decreases, characteristic absorption bands of NDI^2–^ at 395 and 418 nm (Figure 3c) start to grow until all previously formed NDI^•–^ are converted. Finally, upon continued cathodic potential scan, all previously accumulated PMDI^•–^ are transformed into PMDI^2–^, which absorbs around 519 and 551 nm (Figure 3d). All of the above assignments are assisted by spectroelectrochemistry measurements on monolinker Zn(NDI) and Zn(PMDI) thin films (Figures S16–S19).^21^ Importantly, the stepwise transformation of different electronic states in the cathodic scan can be perfectly reversed during the anodic back scan (Figure S15), demonstrating that the mixed-linker MOF can be reversibly switched between up to five distinct redox states by modulating the applied potential. For an easy comparison of these redox states, their representative spectroscopic signatures are summarized in Figure 4a. In addition to its native neutral state Zn(NDI)_0.5_(PMDI)_0.5_, four more charged electronic states are accessible with excellent spectroscopic resolution: the singly reduced monoradical state Zn(NDI^•–^)_0.5_(PMDI)_0.5_, doubly reduced mixed radical state Zn(NDI^•–^)_0.5_(PMDI^•–^)_0.5_, 3-fold reduced mixed radical-dianion state Zn(NDI^2–^)_0.5_(PMDI^•–^)_0.5_, and 4-fold reduced mixed dianion state Zn(NDI^2–^)_0.5_(PMDI^2–^)_0.5_. Most importantly, the appearance of these electronic states is controlled by the applied potential. With absorption features at 360, 471, 713, 418, and 551 nm that are unique for the five electronic states (Figure 4a), their evolution as a function of the applied potential is presented in Figure 4b. Clearly, the transformations are highly sequential and appear in the order of NDI/NDI^•–^, PMDI/PMDI^•–^, NDI^•–^/NDI^2–^, and PMDI^•–^/PMDI^2–^ along the cathodic potential modulation (Figure 4b), which is in line with the electrochemistry data in Figure 2c. Operando spectroelectrochemistry studies of the mixed-linker thin films of different compositional ratios are presented in Figures S20–S25.

### Charge Propagation in the Mixed-Linker Thin Films Monitored by Spectrochronoamperometry

**Figure 5:**
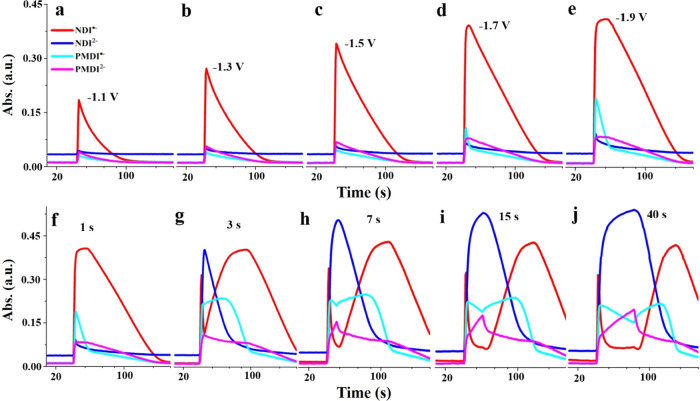
Short-pulsed (1 s) step-potential spectrochronoamperometry monitoring the evolution of NDI^•–^ (471 nm), NDI^2–^ (418 nm), PMDI^•–^ (713 nm), and PMDI^2–^ (551 nm) after stepping the potential from −0.2 to −1.1 V (a), −1.3 V (b), −1.5 V (c), −1.7 V (d), and −1.9 V (e) vs Ag/AgNO_3_, followed by open circuit operation up to 300 s. To activate dianions, a constant step-potential from −0.2 to −1.9 V vs Ag/AgNO_3_ was performed while the pulse was prolonged from 1 s (f) to 3 s (g), 7 s (h), 15 s (i), and 40 s (j).

So far, five different states of the mixed-linker thin films were accessed in CV experiments and further confirmed by their unique optical signatures. However, the modes of electron propagation through the MOFs are still unclear, as two mechanistically different pathways are potentially feasible in the multivariate MOFs: diffusional electron transport between linkers of the same type (hereafter called homolinker hopping) and thermodynamically driven electron transfer between linkers of different types (heterolinker electron transfer). CV experiments, in particular, at slow scan rates, are unsuitable to discriminate between the two processes, as the two NDI-based and the two PMDI-based reductions are well separated in potential. At slow scan rates, the finite diffusion limits of the CV experiments are explored, and all NDIs will be reduced to NDI^•–^ before any PMDI^•–^ is reduced. Consequently, no PMDI^•–^-to-NDI electron transfer can be expected at slow scan rates, as all NDI linkers (the potential acceptor) have already been reduced to their NDI^•–^ state at the potential when PMDI^•–^ is produced. The MOF thin films were thus investigated by short-pulsed (1s) cathodic step-potential chronoamperometry (followed by open circuit operation up to 300 s). The response kinetics of the films were monitored by operando UV–vis spectroscopy at the signature wavelengths of the four reduced linker states (NDI^•–^, PMDI^•–^, NDI^2–^, and PMDI^2–^). Figure 5a–e depicts the time-resolved optical responses of the films and the build-up and decay of the NDI^•–^, PMDI^•–^, NDI^2–^, and PMDI^2–^ states after stepping the potential for 1 second to −1.1, −1,3, −1.5, −1.7, and −1.9 V vs Ag/AgNO_3_. The experiments were conducted on one electrode, allowing the comparison of the signal amplitudes of each linker state between the different spectrochronoamperometry experiments.

At an applied potential of −1.1 V, only the one-electron reduction of the NDI linker is thermodynamically feasible, and thus, the only signal that increases is that of NDI^•–^ (red trace in Figure 5a). No other species can thermodynamically be involved, and charge propagation through the film has to occur by diffusional homolinker hopping. This diffusional process is characterized by its intrinsic *D*_e_^app^ (vide infra). Important to note is that the film remains in the semi-infinite diffusion regime during the 1 second potential step,^5^ which implies that NDI linkers that are remote from the electrode/MOF interface remain in their neutral state. After the 1 s pulse, the NDI^•–^ decays slowly in the open circuit stage to restore the neutral film. Interestingly, stepping the potential to −1.3 V (Figure 5b) and −1.5 V (Figure 5c) produces exclusively the NDI^•–^, even though PMDI reduction would thermodynamically also be feasible at these potentials. What changes, however, is the magnitude of NDI^•–^ formation, and it thus seems that there is a new “channel” for NDI reduction that has opened at these applied potentials. The additional pathway could be supplied by transiently reduced PMDI^•–^, which is reducing enough to drive additional NDI reductions. As a result, the NDI^•–^ signal increases and the steady-state concentration of the PMDI^•–^ remains low and under the detection limit of the experiment.

**Scheme 1:**
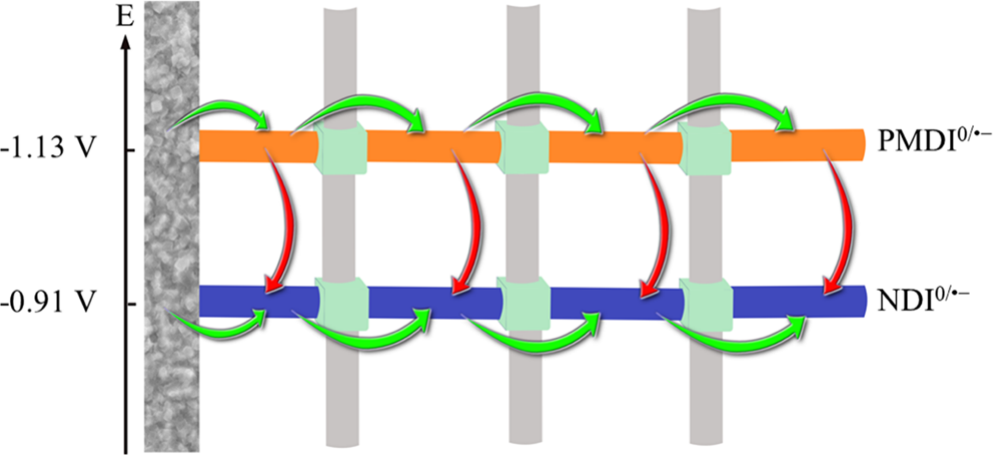
Schematic Illustration of Two Modes of Electron Propagation in the Mixed-Linker MOF: Thermoneutral, Diffusional Homolinker Electron Hopping between Identical Linkers (Green Arrows), and Thermodynamically Driven Heterolinker Electron Transfer (Red Arrows) The involved linkers are organized by their reduction potentials to illustrate the driving force for PMDI^•–^-to-NDI electron transfers. The graphic illustration should not be mistaken as representing a segregation of the linkers in domains.

As the cathodic step-potential is set to −1.7 V vs Ag/AgNO_3_, the simultaneous formation of both NDI^•–^ and PMDI^•–^ can be observed (Figure 5d). The concentration of PMDI^•–^ reaches its peak within the 1 second pulse, while the formation of NDI^•–^ shows an unexpected two-stage growth behavior, a fast phase in the first 1–1.5 s followed by a slow phase up to 3.5 s, thus extending well into the open circuit operation region. This behavior can be better observed upon a further decrease of the step-potential to −1.9 V, where after the fast formation of NDI^•–^ and PMDI^•–^, the slow formation stage of NDI^•–^ extends for almost 10 s (Figure 5e). Afterward, the NDI^•–^ signal plateaus out, indicating exhaustive NDI reduction. Obviously, this prolonged NDI^•–^ formation stage during open circuit operation closely relates to the presence of PMDI^•–^ in the mixed-linker thin film. On one hand, the slow formation stage is missing when PMDI^•–^s are not involved at milder step-potentials (Figure 5b,c), and, on the other, the duration of such slow formation stage overlaps very well with the lifetime of generated PMDI^•–^ at more cathodic step-potentials (Figure 5d,e). Notably, the accumulated NDI^•–^ will not decay until the complete reoxidation of PMDI^•–^. To explain these observations, there must exist a direct PMDI^•–^ to the NDI heterolinker electron-transfer channel so that the presence of the former would effectively protect NDI^•–^ from reoxidation (Scheme 1).

During the short-pulsed (1s) cathodic step-potential chronoamperometry experiments, only the one-electron reduced radical anions of the two linkers are involved. To activate the dianions, a constant step-potential of −1.9 V vs Ag/AgNO_3_ was selected while the pulse was gradually prolonged so that more and more electrons are injected into the mixed-linker MOF thin films (selected examples are shown in Figure 5f–j; for a more systematical comparison, see Figures S26–S29). Indeed, two kinds of dianion species, in the sequence of NDI^2–^ and PMDI^2–^, are observed with distinct formation and disappearance kinetics. This is related to the fact that the evolution of both species is closely intertwined with their respective radical anions, NDI^•–^ and PMDI^•–^. More specifically, the formation and disappearance of NDI^2–^ are accompanied by the decay and growth of NDI^•–^, respectively. An analogous behavior is observed for the PMDI dianion and the radical species. This stepwise electron-transfer mechanism can be further highlighted by serial step-potential reduction and oxidation experiments (Figures S30 and S31). Another important feature in Figure 5f–j is that the appearance of NDI^•–^, PMDI^•–^, NDI^2–^, and PMDI^2–^ signal plateaus is highly sequential, following their thermodynamic ordering. In addition, the sequence of their complete deactivation is reversed, which is further evidenced by the deactivation experiments at different applied potentials (Figures S32–S35). All of these kinetic spectroscopic features point to the fact that in addition to the homolinker redox hopping, heterolinker electron-transfer channels where electrons can flow from more reducing linker states to a less reducing linker must be considered (Scheme 1). Such heterolinker electron transfers have been proposed in the literature on thermodynamic grounds^27,36^ but, to the best of our knowledge, not been proven spectroscopically.

### Tunable Transient-State *D*_e_^app^ in the Mixed-Linker Thin Film

**Figure 6:**
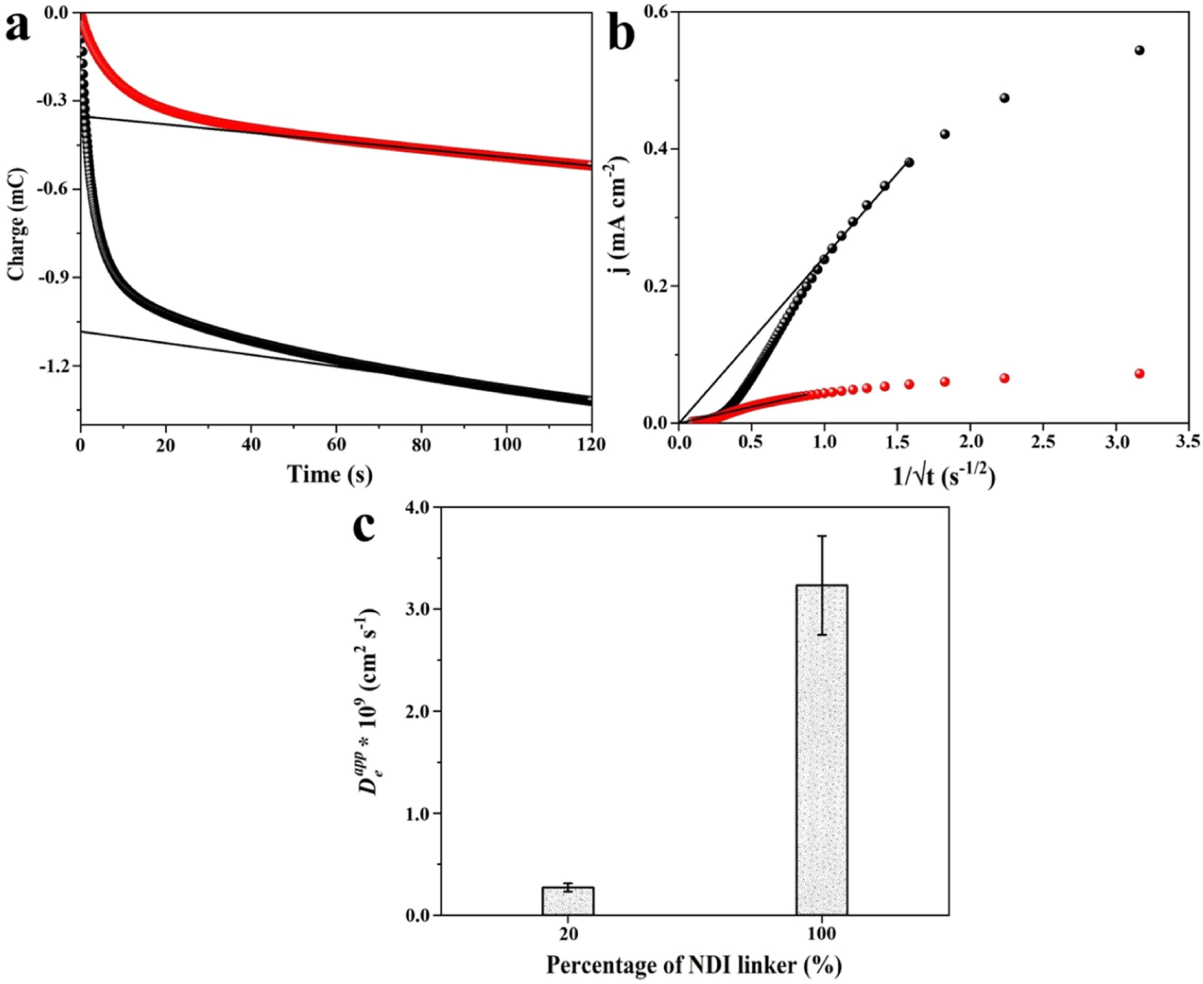
Representative chronocoulometric analysis (a), Cottrell plot (b), and calculated *D*_e_^app^ (c) of Zn(NDI) (black spheres) and Zn(NDI)_0.2_(PMDI)_0.8_ (red spheres) thin films after stepping the potential from −0.2 to −1.0 V vs Ag/AgNO_3_ to reduce NDI linkers to NDI^•–^. The amount of electrochemically addressable NDI linkers was determined by a linear fit to subtract the capacitive contribution. Before each step-potential operation, thin films are poised for at least 60 s at −0.2 V vs Ag/AgNO_3_ to ensure a neutral steady state. All electrochemistry data were collected in Ar-saturated DMF with KPF_6_ as the supporting electrolyte (0.1 M).

An aspect that has not been addressed in the mixed-linker MOFs thus far is that the different linker ratios also give rise to different average distances between linkers of the same type. For MOFs that promote redox hopping between electronically isolated redox-active units, a number of reports have shown that the cation-coupled electron diffusion rate, as characterized by the apparent electron diffusion coefficient, *D*_e_^app^, is depending on the distance between the hopping sites.^17,28,29^ This transient-state redox hopping property is experimentally evaluated from chronoamperometry or spectrochronoamperometry, where a substantial current or absorption response will be observed after a large potential step.^22,28,29,35,37,38^ Here, we compared the *D*_e_^app^ values for charge diffusion through 20 and 100% NDI thin films at a potential that involves exclusively the NDI^•–^ state. After applying a potential positive of the NDI/NDI^•–^ couple for 60 s (to ensure all NDI linkers are in a neutral state), the potential was stepped to an appropriately negative potential to reduce NDI linkers to NDI^•–^ while not reducing PMDI linkers. Chronocoulometric analysis was performed in order to quantify the amount of electrochemically addressable NDI linkers in the MOF thin films (Figure 6a), from which the molar concentration of associated electroactive species, *C*^0^ (mol cm^–3^), can be calculated (see the Experimental Section for details). In a semi-infinite diffusion regime, the Cottrell relationship of the time-dependent current density, *j*(*t*), will be linear to √*t*, and the slope of such a plot (Figure 6b) can be used to calculate the *D*_e_^app^ according to the following expression
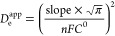
The *D*_e_^app^ of Zn(NDI) was determined to be ∼3.2 × 10^–9^ cm^2^ s^–1^ and is independent of the film thicknesses (films with ∼500 nm to 1 μm thickness), which correlates well with the employed theoretical model. The lower abundance of NDI linker in Zn(NDI)_0.2_(PMDI)_0.8_ led to a significant decrease of *D*_e_^app^ by about 1 order of magnitude (2.7 × 10^–10^ cm^2^ s^–1^; Figure 6c). Apparently, such a reduction in *D*_e_^app^ is the result of increased homolinker redox hopping distances between NDI linkers in the mixed-linker MOF.

### Tunable Steady-State Redox Conductivity in the Mixed-Linker Thin Film

**Figure 7:**
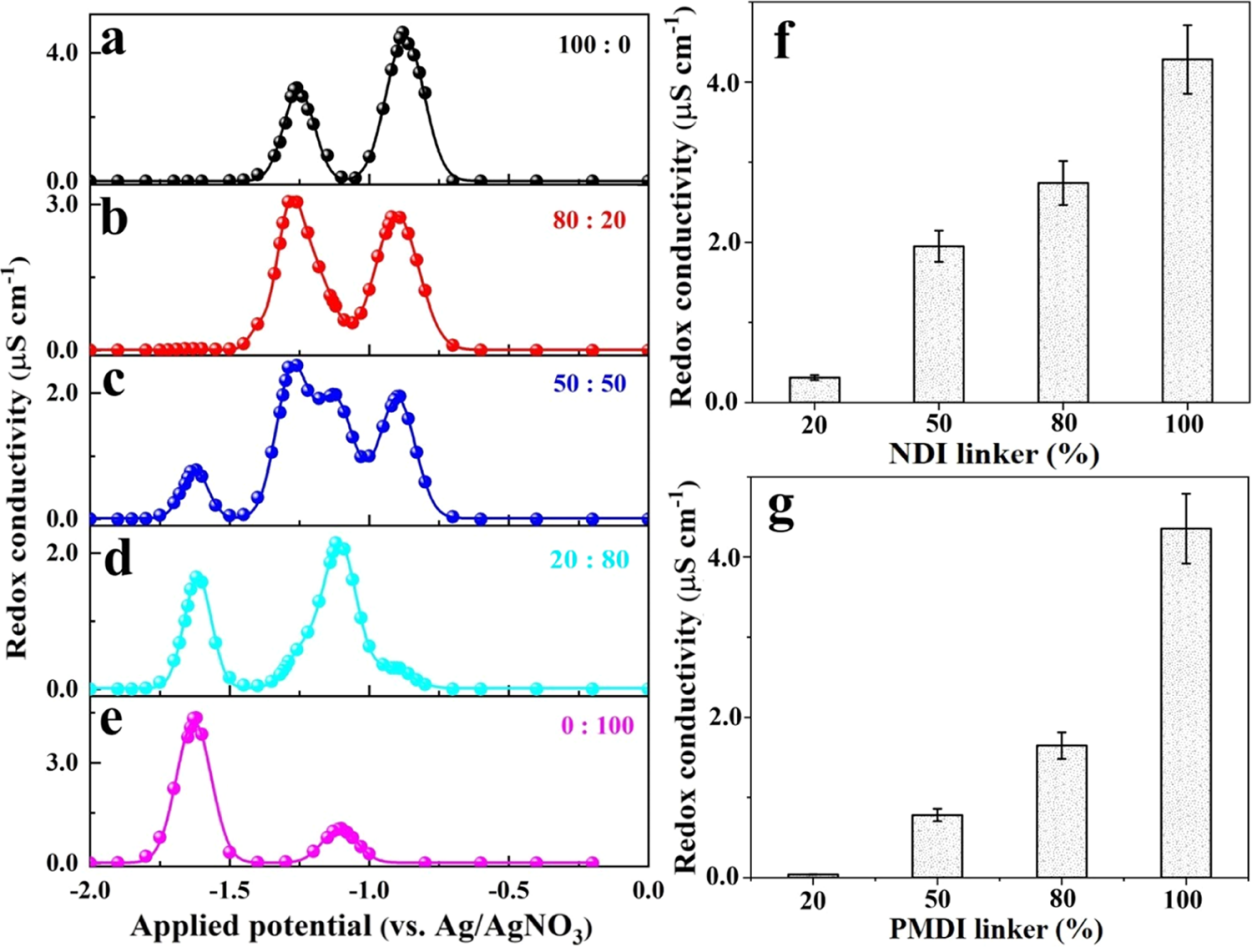
Evolution of steady-state thin film conductivity Zn(NDI) (a), Zn(NDI)_0.8_(PMDI)_0.2_ (b), Zn(NDI)_0.5_(PMDI)_0.5_ (c), Zn(NDI)_0.2_(PMDI)_0.8_ (d), and Zn(PMDI) (e) as a function of applied potential; Gaussian fit was performed for the bell-shaped redox conductivities. Maximum steady-state conductivity corresponding to NDI/NDI^•–^ (f) and PMDI^•–^/PMDI^2–^ (g) redox pairs as a function of the respective linker percentage. All electrochemistry data were collected in Ar-saturated DMF with KPF_6_ as the supporting electrolyte (0.1 M).

The dependence of *D*_e_^app^ on the redox hopping distance thus follows the expected trend, while whether this transient-state redox hopping property can be translated to a steady-state experiment is still unknown. Herein, we present the first study of how the hopping distance would influence the steady-state redox conductivity. Therefore, all monolinker and mixed-linker MOF thin films were studied by steady-state impedance spectroscopy, as recently reported (see the Experimental Section and SI for more details; equivalent circuits and related fitting results are shown in Figures S36–S41).^18^ In analogy to the two distinct bell-shaped redox conductivity profiles resolved for a monolinker Zn(NDI) thin film (Figure 7a),^18^ the monolinker Zn(PMDI) thin film also exhibits two bell-shaped redox conductivity profiles but centered at different potentials (Figure 7e). This is consistent with the differences in formal potentials of the underlying PMDI/PMDI^•–^ and PMDI^•–^/PMDI^2–^ redox couples. Interestingly, while the maximum redox conductivity in the Zn(NDI) thin film is observed at an applied potential of the first reduction, i.e., the NDI/NDI^•–^ couple, it is rather low at the corresponding PMDI/PMDI^•–^ couple of the Zn(PMDI) thin film. In fact, the redox conductivity of the Zn(PMDI) film is highest at the applied potential of the PMDI^•–^/PMDI^2–^ redox pair. This phenomenon is highly reproducible but not entirely understood at present. Intriguingly, the low redox conductivity centered around the PMDI/PMDI^•–^ couple is paralleled by an unusually large peak-to-peak separation of this couple in the CV of the Zn(PMDI) film (Figure 2e). Both of these phenomena may be caused by unusually slow rates of cation-coupled electron transport, which may be caused by unusually high reorganization energies or strong PMDI^•–^···K^+^ ion pair interactions. Whatever the reason may be, none of these effects are observable any longer in the Zn(NDI)_0.2_(PMDI)_0.8_ films with 20% NDI linker. For the mixed-linker Zn(NDI)_0.5_(PMDI)_0.5_, four distinct redox conductivity features can be observed with decent peak separations (Figure 7c). Compared to the monolinker counterparts, this merged redox conductivity profile in the mixed-linker MOF significantly expands the operational potential window, enabling a variety of applications, notably in energy storage and electrocatalysis.^16,24^ More importantly, a profound effect of the linker ratio on the experimental redox conductivities is manifested (Figure 7b–d). Specifically, the maximum conductivity corresponding to the NDI/NDI^•–^ redox pairs shows a clear dependence on the homolinker hopping distances (Figure 7f). The maximum conductivity drops from ∼4.3 μS cm^–1^ of the monolinker Zn(NDI) thin films to only ∼0.3 μS cm^–1^ in Zn(NDI)_0.2_(PMDI)_0.8_, a more than 10-fold decrease. This greatly retarded electric conductance at high NDI to NDI distances aligns with the percolation theory. In fact, the maximum conductivity changes corresponding to the PMDI^•–^/PMDI^2–^ redox pairs align even better with the percolation theory, with more than 2 orders of magnitude reduction when the PMDI linker ratio decreases from 100 to 20% (Figure 7g). This means that the statistically distributed NDI and PMDI linkers are acting as dielectric spacers for each other at steady-state potentials. By simply controlling the ratio of the two linkers, redox conductivities of the mixed-linker MOFs can systematically be tuned. Essentially, this has introduced a new strategy to tune the steady-state redox conductivity on top of its potential dependency.

### Transient-State vs Steady-State Redox Hopping in the Mixed-Linker Thin Films

While the *D*_e_^app^ and the redox conductivity in the mixed-linker MOFs both show a hopping distance dependence, the conditions under which the two processes are addressed are fundamentally different. The *D*_e_^app^ is obtained from transient chronoamperometry experiments, which involve a large step-potential and probes the diffusional redox hopping that requires the ingress of charge balancing counterions. The steady-state conductivity measurement by impedance spectroscopy, in contrast, neither requires the ingress/egress of charged ions nor is of diffusional character. Nevertheless, the two processes rely on the identical elementary step for charge propagation, that is, the cation-coupled self-exchange between a reduced and an oxidized linker.^18,22^ This means that any modulations of this elementary step can affect both transient and steady states. Last but not least, we want to highlight that the steady-state redox conductivity is highly dependent on the applied potentials, as evidenced by the bell-shaped conductivity curves presented in Figure 7a–e. This fact implies that the steady-state redox conductivity is not a constant value but can differ by up to 4 orders of magnitudes depending on the film redox state.^18^ The transient-state *D*_e_^app^, however, is generally assumed to be a constant value, representing the purely diffusional nature of the redox hopping process. Considering these differences, it is clear that the redox conductivity of an MOF cannot be calculated simply from its *D*_e_^app^, and one should keep the *D*_e_^app^ from transient experiments separated from the steady-state redox conductivity measurements.

## Conclusions

In summary, a multivariate MOF containing two statistically distributed redox-active linkers, NDI and PMDI, is reported. The proportion of the two linkers in the MOF can be customized through variations of the feeding ratio during the solvothermal synthesis. The mixed-linker MOFs feature four distinct and reversible redox waves that are assigned to the NDI/NDI^•–^, PMDI/PMDI^•–^, NDI^•–^/NDI^2–^, and PMDI^•–^/PMDI^2–^ redox couples of the constituting linkers. These assignments are supported by characteristic spectroscopic signatures of each neutral, anion radical, and dianion species and probed at operando conditions. Pulsed step-potential spectrochronoamperometry reveals two channels for electron propagation through the MOF thin films: the thermoneutral, homolinker electron hopping and the thermodynamically downhill heterolinker electron-transfer channel. From an application viewpoint, systems such as the mixed-linker MOFs presented herein provide facile electron transport to acceptor sites that are deeply buried within an MOF but without being in direct contact with the environment. This situation mimics that in redox-active enzymes like hydrogenases^39^ and provides unexplored opportunities in mediated redox chemistry in the interior of MOFs.

The rate of homolinker hopping transport, as characterized by the apparent electron diffusion coefficient, *D*_e_^app^, exhibits the expected distance dependence between the hopping sites. Interestingly, a similar distance dependence is also observed for the maximum redox conductivity under steady-state conditions, highlighting that cation-coupled electron self-exchange between neighboring redox-active sites is the mutual elementary step in both experiments. The four redox events in the mixed-linker MOFs give rise to four distinct potential-dependent steady-state redox conductivity features, which significantly extend the working window of such redox-conductive materials with potential applications, for example, in field-effect transistors.

## Experimental Section

### Synthesis of Mixed-Linker MOF Thin Film on FTO Surface

The synthesis of the NDI and PMDI linkers follows previously published protocols,^32^ and their purity was confirmed by ^1^H NMR spectroscopy. FTO substrates were cut into 1 cm × 2 cm plates and cleaned successively in solutions of deionized water, ethanol, and acetone by sonication for 20 min each. Cleaned FTO substrates were placed in a 20 mL scintillation vial with the conductive side facing down. To this vial, predissolved NDI linker (2.75 mmol L^–1^), PMDI linker (2.75 mmol L^–1^), and Zn(NO_3_)_2_·6H_2_O (6.65 mmol L^–1^) in DMF solutions were added. The ratio between NDI to PMDI was tuned ranging from 100/0, 80/20, 50/50, 20/80, to 0/100 by adjusting the volume of each linker solution used, and the Zn to linker ratio was kept at 1.1:1. Afterward, the vial was sealed, placed into an oven, heated slowly from room temperature to 135 °C (heating rate 5 °C/min), and held at this temperature for 3.5 h. After that, the reaction mixture was slowly cooled to room temperature, and the obtained mixed-linker thin films were washed with DMF. The unwanted growth on the glass side of the FTO was carefully removed by Kimtech wiping paper wetted with DMF. Subsequently, the cleaned thin films were sonicated in DMF for 10 s to remove loosely bonded particles. Finally, the mixed-linker thin films were thoroughly washed with DMF and stored in DMF for future use.

### Electrochemistry and UV–Vis Spectroelectrochemistry

All cyclic voltammetry (CV) and chronoamperometry experiments were performed on a Metrohm Autolab potentiostat (PGSTAT302) with Nova 2.1.4 software in a one-compartment, three-electrode configuration, with the mixed-linker thin films on FTO as the working electrode (testing area is around 1 cm^2^), a glassy carbon rod as the counter electrode, and a nonaqueous Ag/Ag(NO_3_) (0.01 M in acetonitrile) as the reference electrode (measured as −0.09 ± 0.02 V vs Fc^+/0^). UV–vis spectroelectrochemistry was conducted by integrating the electrochemistry setup described above with a Varian Cary 50 UV–vis spectrophotometer. A cuvette was employed as the electrochemical cell, and all electrodes remain the same as above except that a Pt rod was employed as the counter electrode. Absorption spectra were collected in kinetic mode along with the electrochemistry operations. All measurements were performed in Ar-saturated DMF solutions using 0.1 M KPF_6_ as the supporting electrolyte.

### Steady-State Redox Conductivity Measurements

The steady-state redox conductivity of the mixed-linker MOF was carried out using electrochemical impedance spectroscopy (EIS) under the same experimental electrochemistry conditions as stated above.^18^ All thin films were equilibrated at designated potentials for at least 30 s before each EIS measurement. A frequency range of 0.1–10000 Hz and a 10 mV AC potential modulation were employed for all measurements. All acquisitioned data were fitted with three different equivalent circuits; they are a simple RC circuit without the diffusional element, modified RC circuit considers specifically a semi-infinite diffusion-related Warburg element, or a constant phase element (see Figure S36 for details). The fitted intersite redox hopping resistances were used to calculate the steady-state redox conductivities at different applied potentials employing Ohm’s law: σ = ι/*RA*, where σ is the steady-state redox conductivity, ι is the thickness of the mixed-linker MOF thin film, *R* is the resistance related to the intersite redox hopping, and *A* is the measurement area of the film.

### Measurements of Transient-State *D*_e_^app^

The transient-state *D*_e_^app^ of the mixed-linker MOF was determined with chronoamperometry experiments using the same electrochemistry conditions as those stated above. Before each chronoamperometry measurement, the thin film was preconditioned at −0.2 V versus Ag/AgNO_3_ for at least 60 s to ensure a neutral steady state. Afterward, the applied potential was stepped up to −1.0 V vs Ag/AgNO_3_ to selectively reduce NDI linkers to NDI^•–^. By plotting the chronoamperometry data in the form of chronocoulometry, and after a linear fit to subtract the capacitive contributions, the amount of electrochemically addressable NDI linkers was determined. Finally, the Cottrell plot of the time-dependent current density, *j*(*t*), with respect to √*t*, was used to determine a slope, which was later used to calculate *D*_e_^app^ according to the Cottrell expression.

## Supporting Information Available

Linker characterizations; MOF thin film characterizations; spectroelectrochemistry and pulsed step-potential spectrochronoamperometry studies; steady-state redox conductivity studies; and other characterization data (PDF)
