# Supplementary material for: Diffusional Electron Transport Coupled to Thermodynamically Driven Electron Transfers in Redox-Conductive Multivariate Metal–Organic Frameworks
Source: J Am Chem Soc. 2024 Apr 19;146(17):12000–10. doi: 10.1021/jacs.4c01401 (PMC11066865; doi:10.1021/jacs.4c01401)
Supplement: Supplementary file 1 — ja4c01401_si_001.pdf [file ja4c01401_si_001.pdf]

Diffusional electron transport coupled to thermodynamically driven electron transfers in redox-conductive multivariate metal-organic frameworks

*Jingguo Li,[1,2] Amol Kumar,[1] Sascha Ott[1,2]**

Sascha.ott@kemi.uu.se

[1]Department of Chemistry - Ångström Laboratory, Uppsala University, Box 523, 75120 Uppsala, Sweden.

[2]Wallenberg Initiative Materials Science for Sustainability, Department of Chemistry - Ångström Laboratory, Uppsala University, Box 523, 75120 Uppsala, Sweden.

Table of Contents

1. Linker characterizations 3

1.1. ^{1}HNMR 3

1.2. UV-vis absorption 5

1.3. Electrochemistry 6

2. MOF thin film characterizations 7

2.1. SEM 7

2.2. Thin film XRD 9

2.3. Electrochemistry 10

2.4. Input and output linker ratio 13

2.5. UV-vis absorption 14

3. Spectroelctrochemistry studies 15

3.1. $Zn(NDI)_{0.5}(PMDI)_{0.5}$ thin film 15

3.2. Zn(NDI) thin film 16

3.3. Zn(PMDI) thin film 18

3.4. $Zn(NDI)_{0.2}(PMDI)_{0.8}$ thin film 20

3.5. $Zn(NDI)_{0.8}(PMDI)_{0.2}$ thin film 23

4. Pulsed step-potential spectrochronoamperometry studies 26

5. Steady-state redox conductivity studies 36

5.1. Equivalent circuits 36

5.2. Zn(NDI) thin film 37

5.3. $Zn(NDI)_{0.2}(PMDI)_{0.8}$ thin film 38

5.4. $Zn(NDI)_{0.5}(PMDI)_{0.5}$ thin film 39

5.5. $Zn(NDI)_{0.8}(PMDI)_{0.2}$ thin film 40

5.6. Zn(PMDI) thin film 41

1. Linker characterizations

1.1. ^{1}HNMR

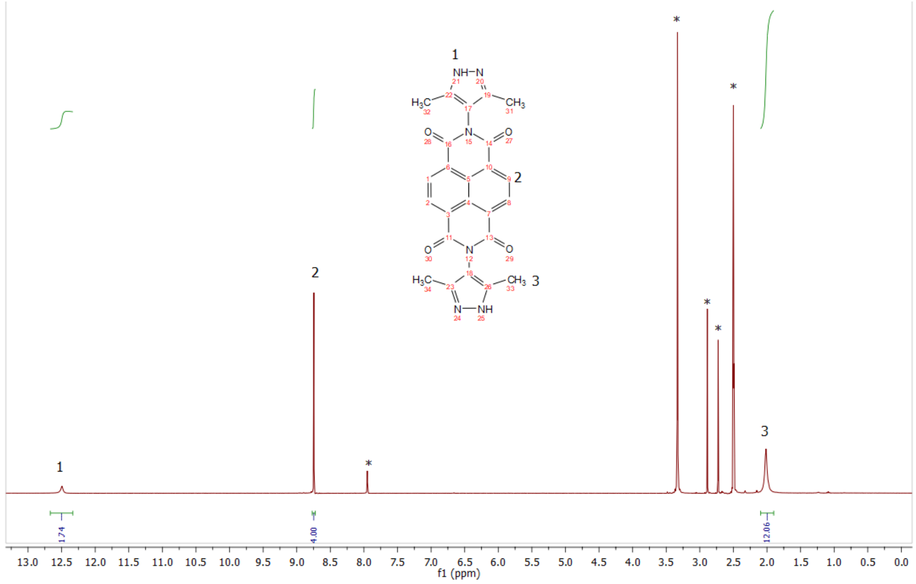

Figure S1. ^{1}HNMR of **NDI** linker measured in DMSO-d_6 at 293 K. The nondeuterated solvent and water are marked with asterisk sign.

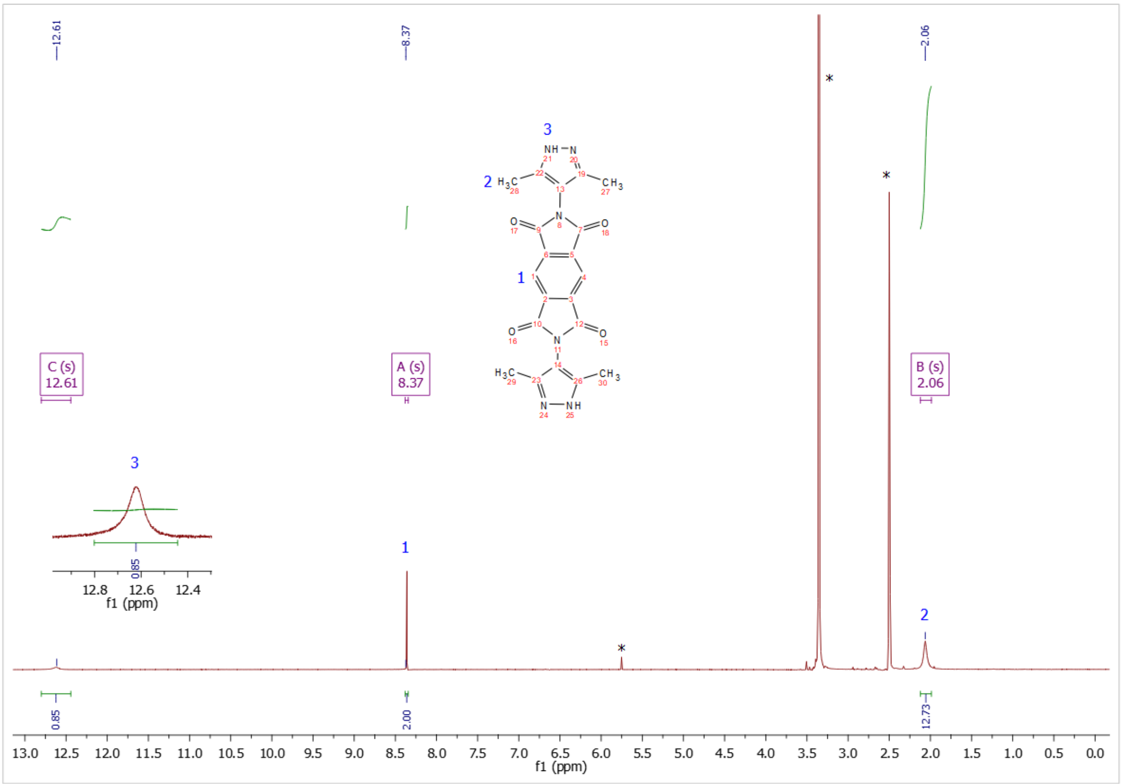

Figure S2. ^{1}H NMR spectrum of **PMDI** in DMSO-d6 at 293 K. Peak at 3.33 ppm corresponds to water, 5.63 ppm corresponds to DCM.

1.2. UV-vis absorption

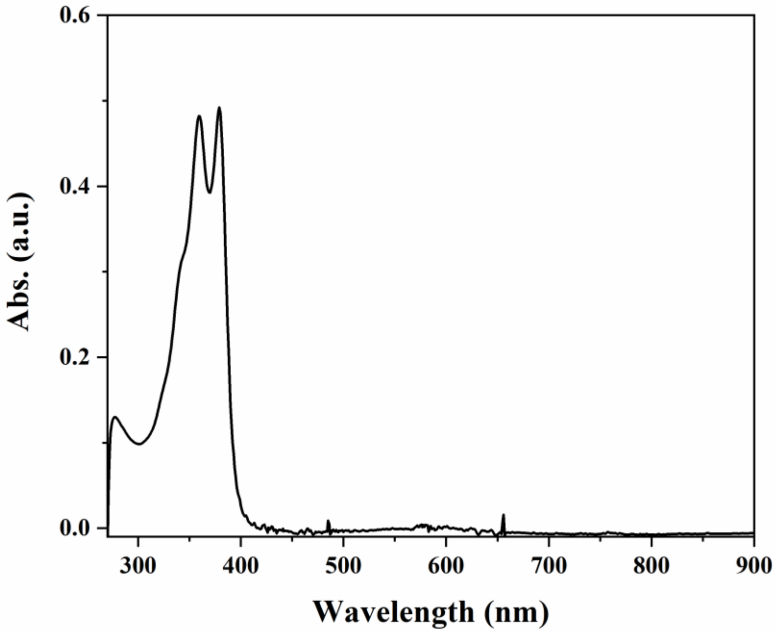

Figure S3. UV-vis absorption spectra of the free NDI linker in DMF, showing that the electronic π–π* transitions in the neutral states. UV-vis absorption of NDI in solution is measured with DMF as reference to avoid any possible contribution from DMF.

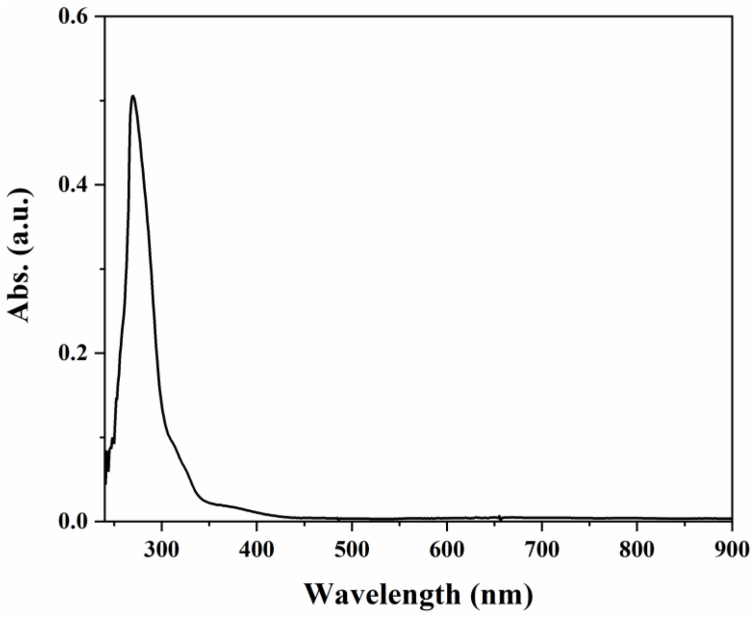

Figure S4. UV-vis absorption spectra of the free PMDI linker in DMF, showing that the electronic π–π* transitions in the neutral states. UV-vis absorption of PMDI in solution is measured with DMF as reference to avoid any possible contribution from DMF.

1.3. Electrochemistry

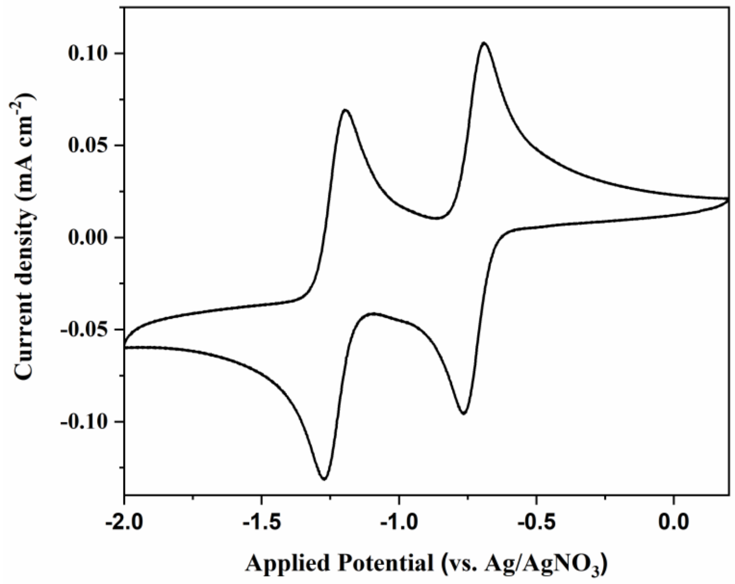

Figure S5. CV of NDI linkers measured at scan rates from 50 mV s^{-1} in DMF with 0.1 M KPF_6 as the supporting electrolyte.

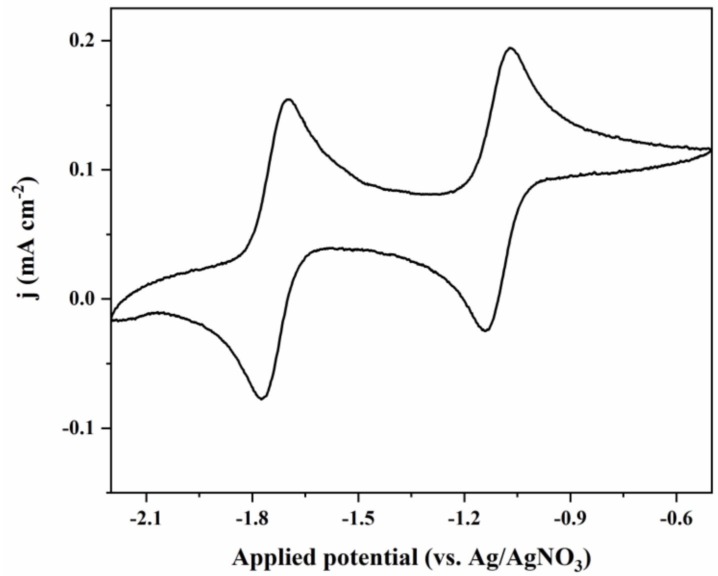

Figure S6. CV of PMDI linkers measured at scan rates from 50 mV s^{-1} in DMF with 0.1 M KPF_6 as the supporting electrolyte.

2. MOF thin film characterizations

2.1. SEM

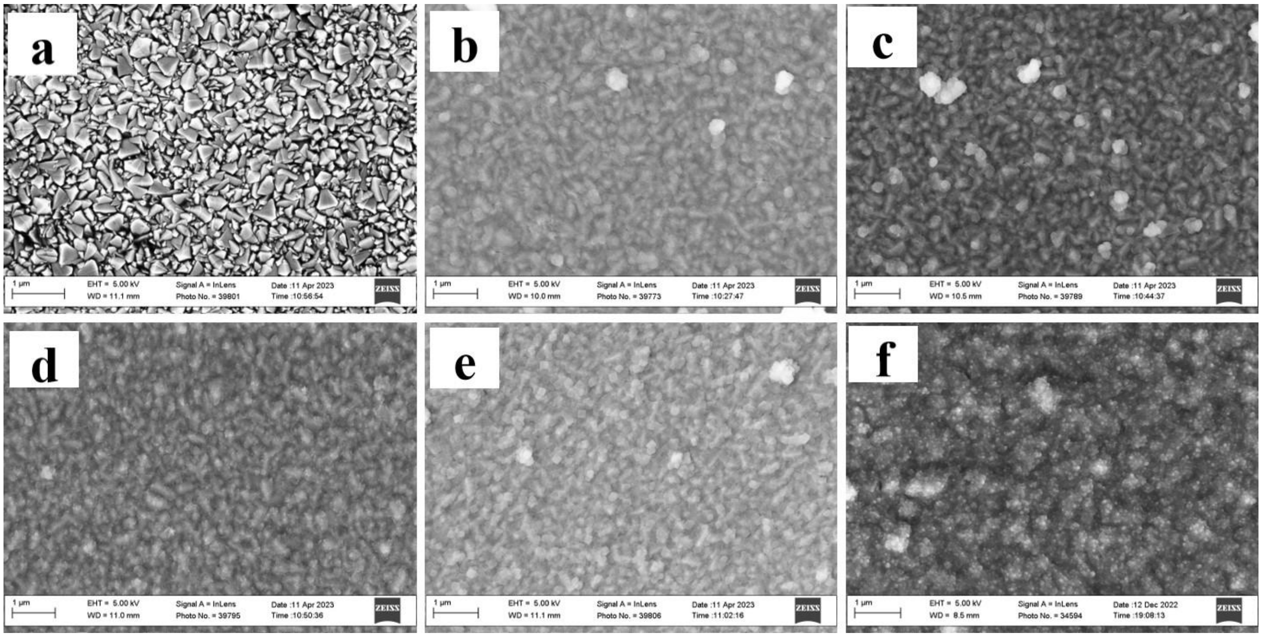

Figure S7. SEM top surface images of the bare FTO (a), and Zn(NDI) (b), Zn(NDI)$_{0.8}$(PMDI)$_{0.2}$ (c), Zn(NDI)$_{0.5}$(PMDI)$_{0.5}$ (d), Zn(NDI)$_{0.2}$(PMDI)$_{0.8}$ (e), Zn(PMDI) (f) thin films on the surface of FTO substrate. Homogeneous and compact top surfaces are constantly observed for MOF thin films while also featuring continuous morphological evolutions as the linker ratio varies. The micro-crystallites are getting smaller and surface morphology becomes rougher as the ratio of PMDI linker increases.

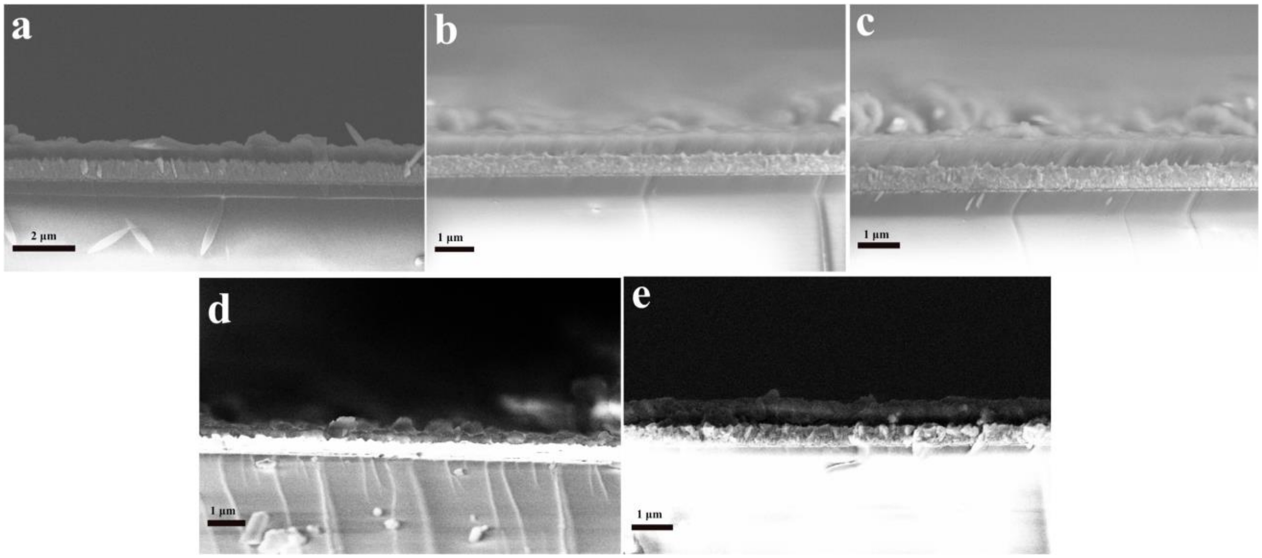

Figure S8. SEM cross-section images of Zn(NDI) (a), Zn(NDI)$_{0.8}$(PMDI)$_{0.2}$ (b), Zn(NDI)$_{0.5}$(PMDI)$_{0.5}$ (c), Zn(NDI)$_{0.2}$(PMDI)$_{0.8}$ (d), Zn(PMDI) (e) thin films on the surface of FTO substrate. Compact film morphologies are constantly observed for all MOF on the FTO surface. The thickness of the thin films is ranging between 500 nm to 800 nm.

2.2. Thin film XRD

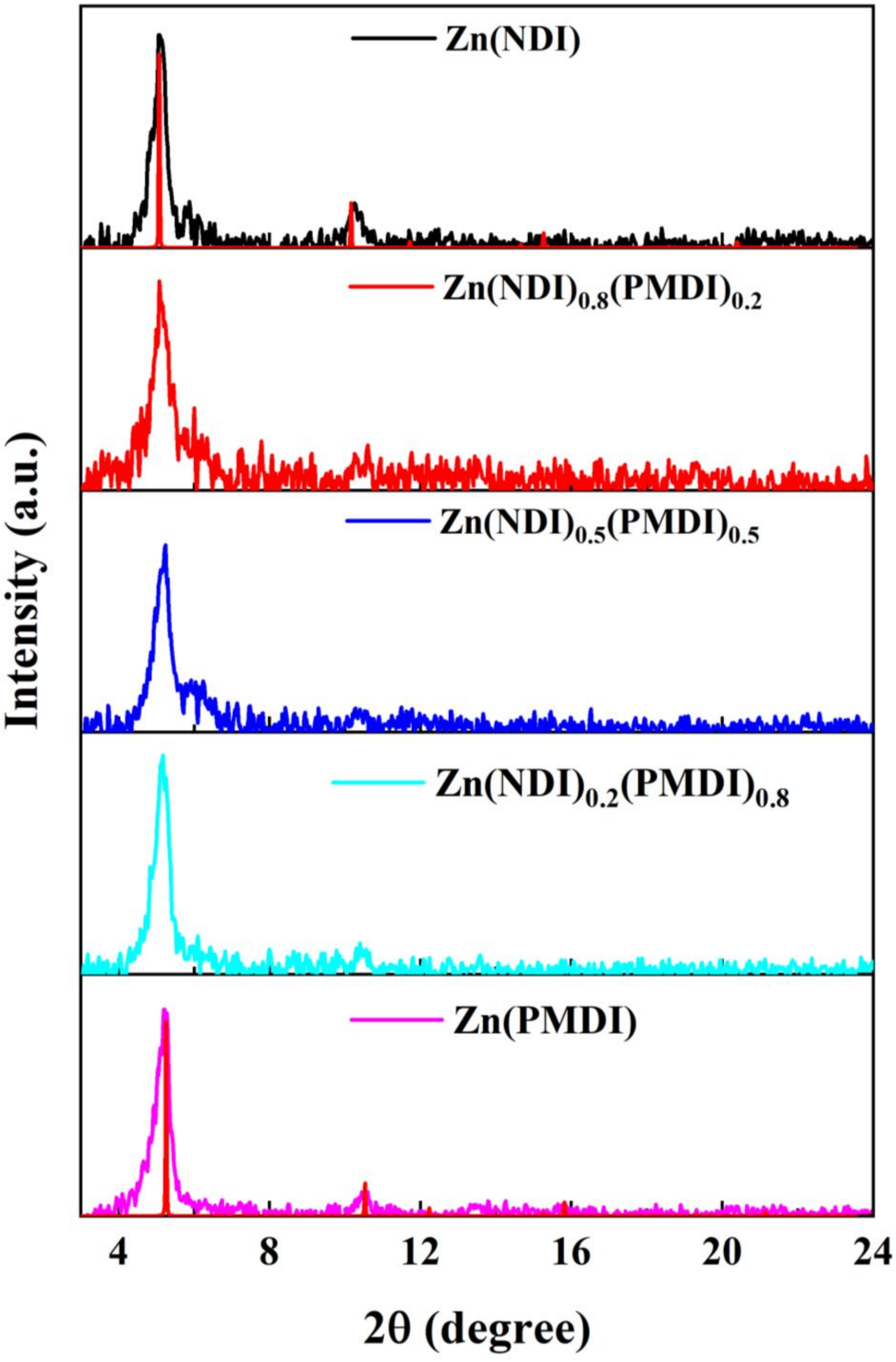

Figure S9. Experimental thin film X-ray diffraction (XRD) data of mono-linker and mixed linker MOF thin films FTO surfaces together with simulated powder XRD data for the mono-linker MOFs.

2.3. Electrochemistry

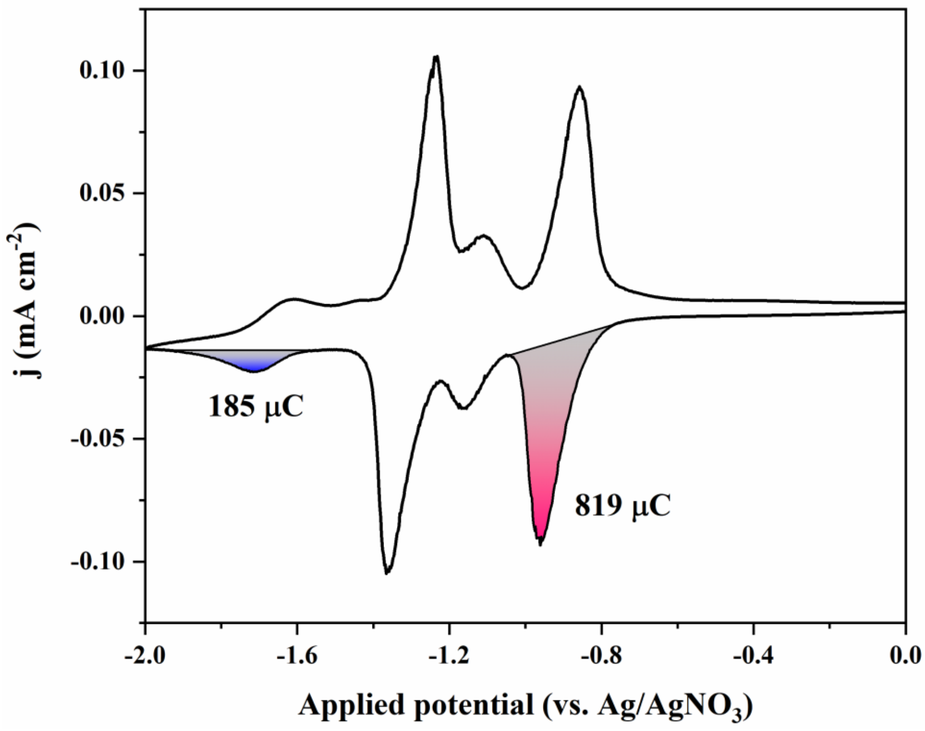

Figure S10. Thin film CV of mixed linker $Zn(NDI)_{0.8}(PMDI)_{0.2}$ on FTO surface with a scan rate of 10 mV s^{-1}. The integrated area was used to determine the ratio of NDI to PMDI in the mixed linker MOFs. All electrochemistry data were collected in Ar-saturated DMF with KPF_6 as the supporting electrolyte (0.1 M).

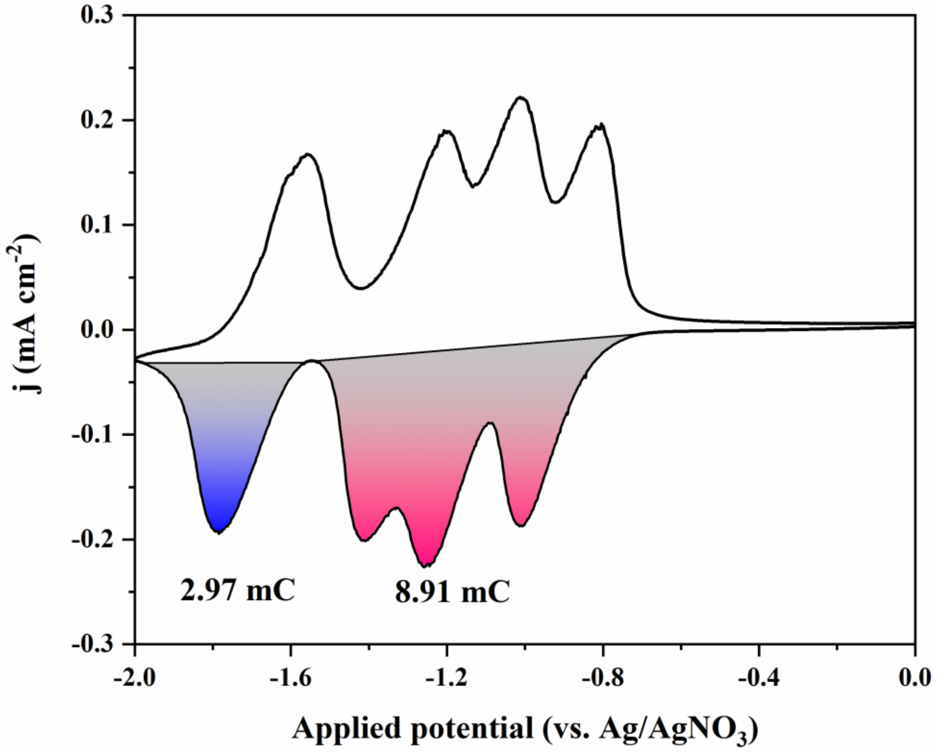

Figure S11. Thin film CV of mixed linker $Zn(NDI)_{0.5}(PMDI)_{0.5}$ on FTO surface with a scan rate of 10 mV s^{-1}. The integrated area was used to determine the ratio of NDI to PMDI in the mixed linker MOFs. All electrochemistry data were collected in Ar-saturated DMF with KPF_6 as the supporting electrolyte (0.1 M).

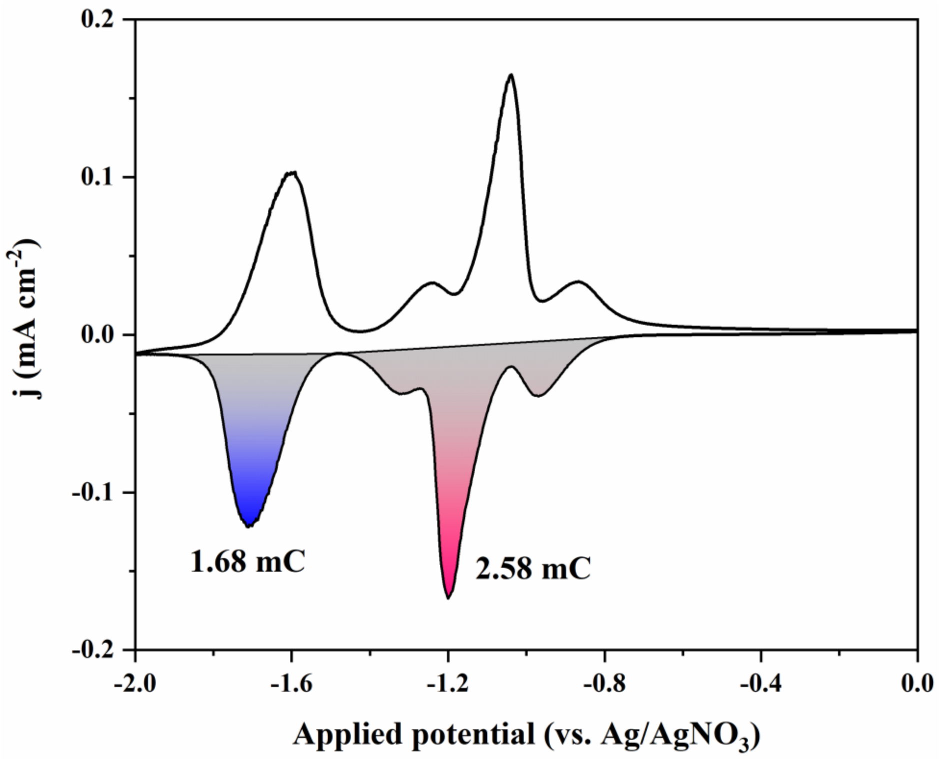

Figure S12. Thin film CV of mixed linker $Zn(NDI)_{0.2}(PMDI)_{0.8}$ on FTO surface with a scan rate of 10 mV s^{-1}. The integrated area was used to determine the ratio of NDI to PMDI in the mixed linker MOFs. All electrochemistry data were collected in Ar-saturated DMF with KPF_6 as the supporting electrolyte (0.1 M).

2.4. Input and output linker ratio

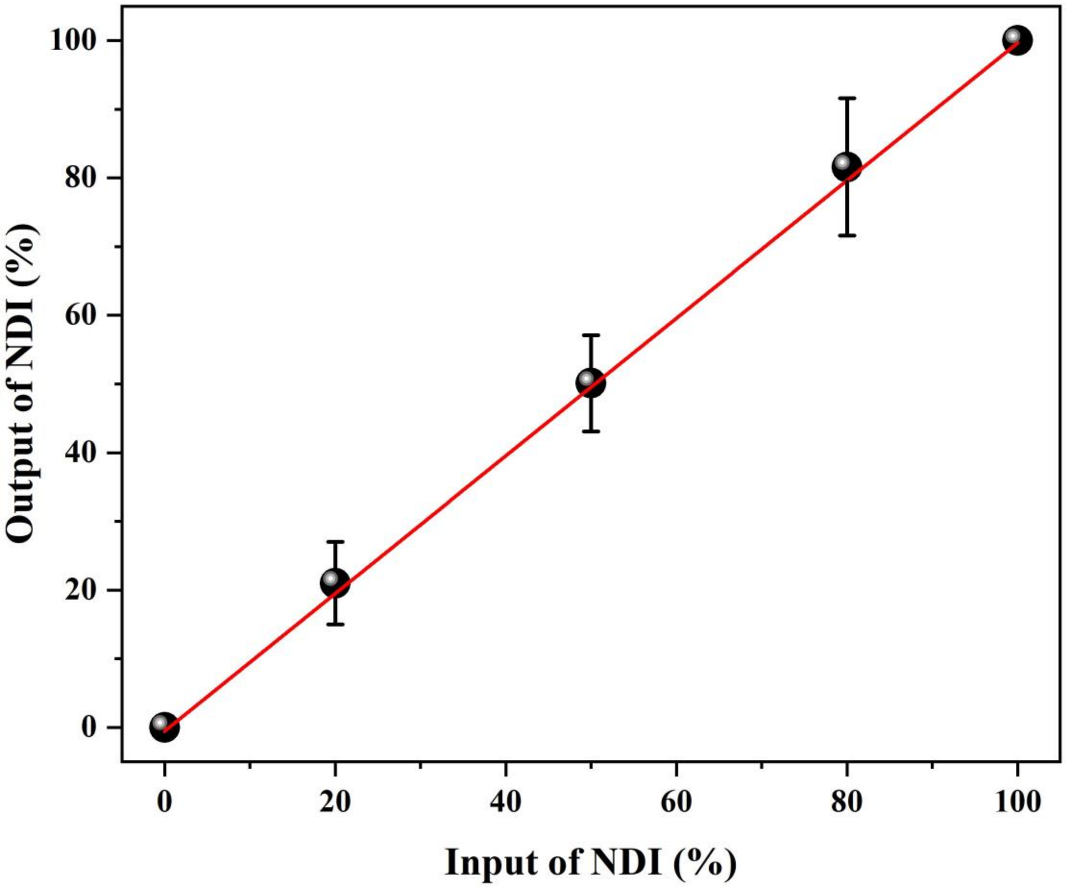

Figure S13. The relation between feeding ratio and actually incorporated ratio of two linkers in the mixed linker MOF as determined by slow scan rate cyclic voltammetry (CV) experiments (**Fig. S9-11**, more discussions below). This excellent correlation makes the chemical composition of the mixed linker MOF programmable in a bottom-up manner.

2.5. UV-vis absorption

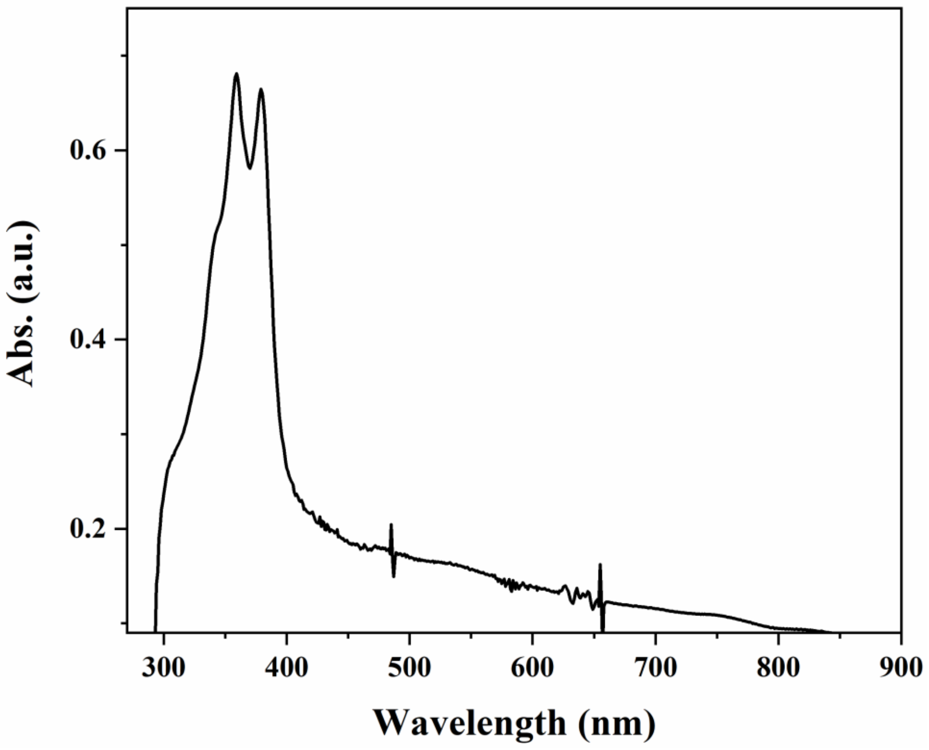

Figure S14. UV-vis absorption spectra of the mono-linker Zn(NDI) thin film on FTO measured in DMF, showing that the electronic π–π* transitions in the neutral states. UV-vis absorption was measured with bare FTO in DMF as reference to avoid any possible contribution from both the electrode and solvent.

3. Spectroelctrochemistry studies

3.1. $Zn(NDI)_{0.5}(PMDI)_{0.5}$ thin film

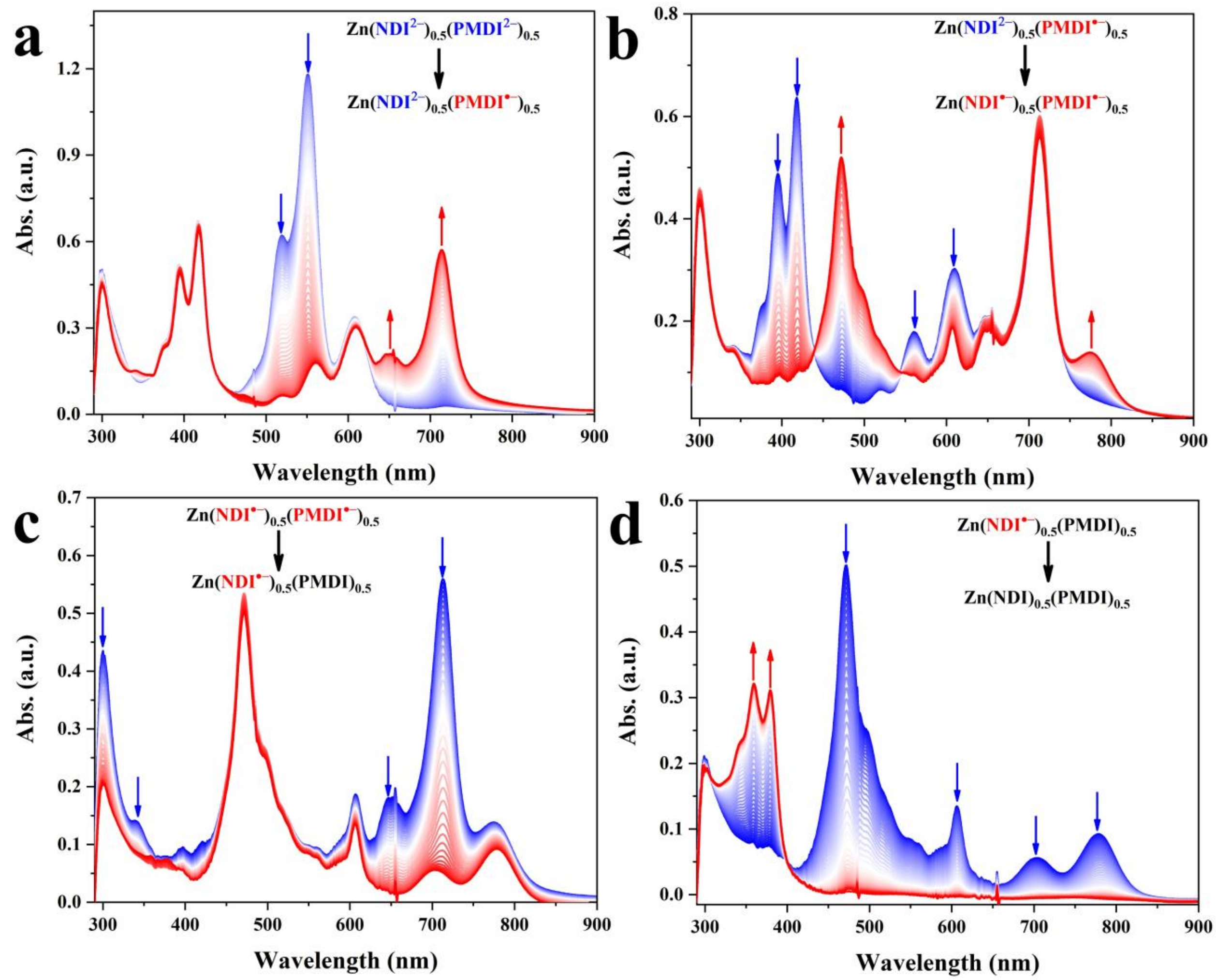

Figure S15. UV-vis spectroelectrochemistry measurements of the $Zn(NDI)_{0.5}(PMDI)_{0.5}$ thin film while slowing re-oxidizing it with finely modulated applied potential to access the vibrational signature of different redox states, from quadruply reduced mixed dianion state $Zn(NDI^{2-})_{0.5}(PMDI^{2-})_{0.5}$ to triply reduced mixed radical-dianion state $Zn(NDI^{2-})_{0.5}(PMDI^{\bullet-})_{0.5}$ (a), then doubly reduced mixed radical state $Zn(NDI^{\bullet-})_{0.5}(PMDI^{\bullet-})_{0.5}$ (b), followed by singly reduced mono-radical state $Zn(NDI^{\bullet-})_{0.5}(PMDI)_{0.5}$ (c), and finally neutral $Zn(NDI)_{0.5}(PMDI)_{0.5}$ (d).

3.2. Zn(NDI) thin film

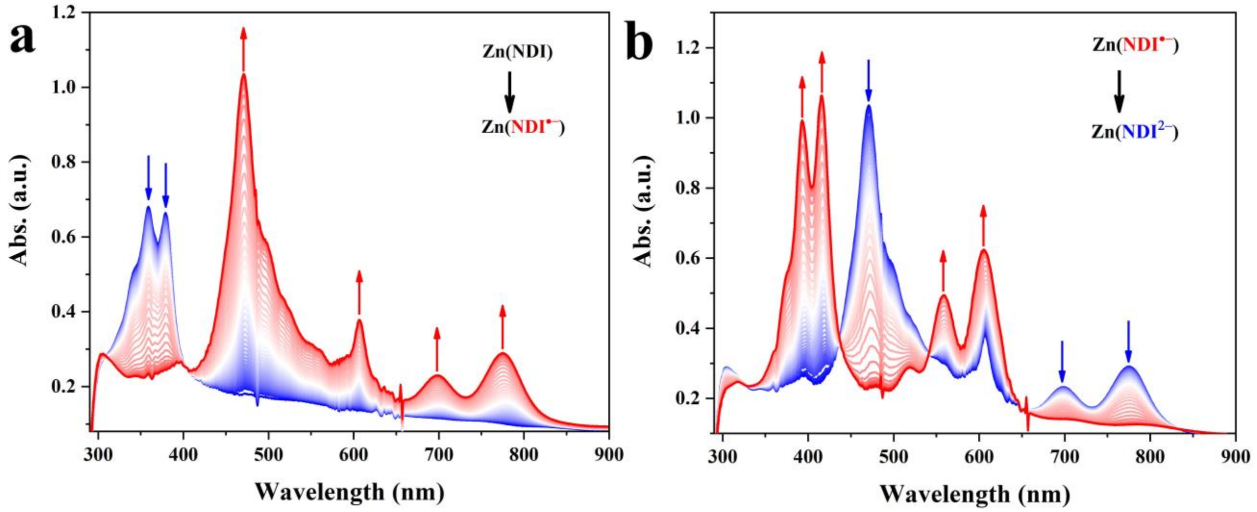

Figure S16. UV-vis spectroelectrochemistry measurements of the Zn(NDI) thin film while slowing reducing it with finely modulated applied potential to access the vibrational signature of different redox states, singly reduced radical state Zn(NDI$^{\bullet-}$) (a) and doubly reduced dianion state Zn(NDI^{2-}) (b).

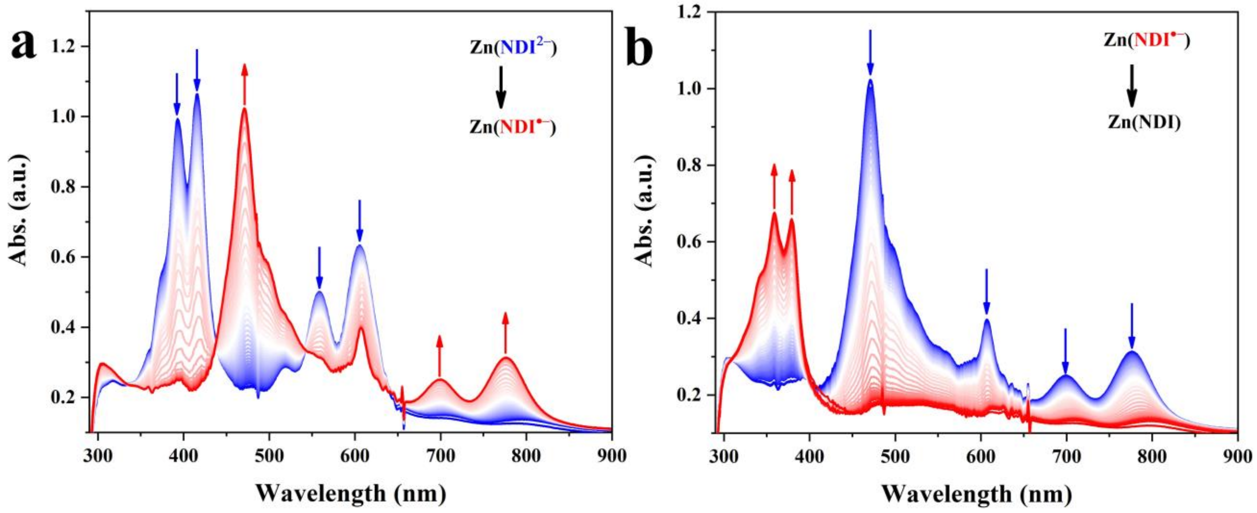

Figure S17. UV-vis spectroelectrochemistry measurements of the Zn(NDI) thin film while slowing re-oxidizing it with finely modulated applied potential to access the vibrational signature of different redox states, from doubly reduced dianion state Zn(NDI^{2-}) to singly reduced radical state Zn($NDI^{\bullet-}$) (a), and further to neutral Zn(NDI) state (b).

3.3. Zn(PMDI) thin film

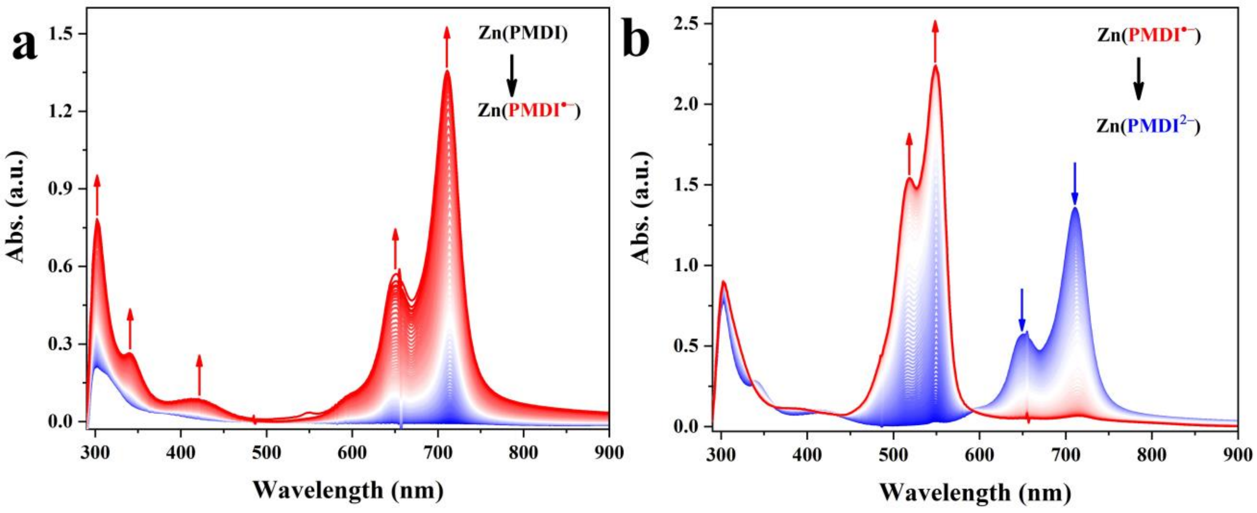

Figure S18. UV-vis spectroelectrochemistry measurements of the Zn(PMDI) thin film while slowing reducing it with finely modulated applied potential to access the vibrational signature of different redox states, singly reduced radical state Zn(PMDI$^{\bullet-}$) (a) and doubly reduced dianion state Zn(PMDI^{2-}) (b).

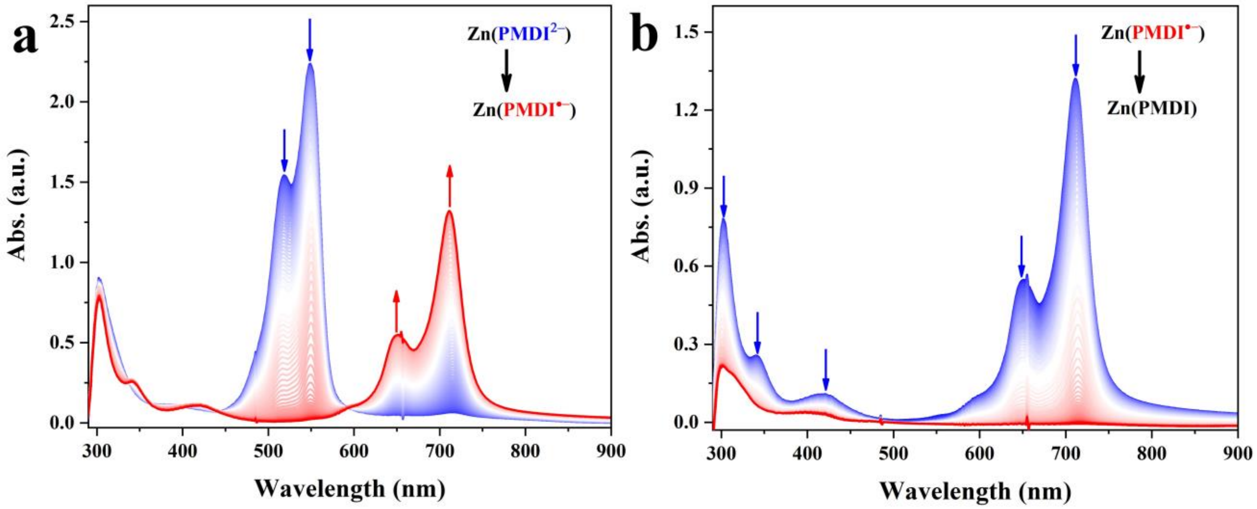

Figure S19. UV-vis spectroelectrochemistry measurements of the Zn(PMDI) thin film while slowing re-oxidizing it with finely modulated applied potential to access the vibrational signature of different redox states, from doubly reduced dianion state Zn(PMDI^{2-}) to singly reduced radical state Zn(PMDI$^{\bullet-}$) (a), and further to neutral Zn(PMDI) state (b).

3.4. Zn(NDI)$_{0.2}$(PMDI)$_{0.8}$ thin film

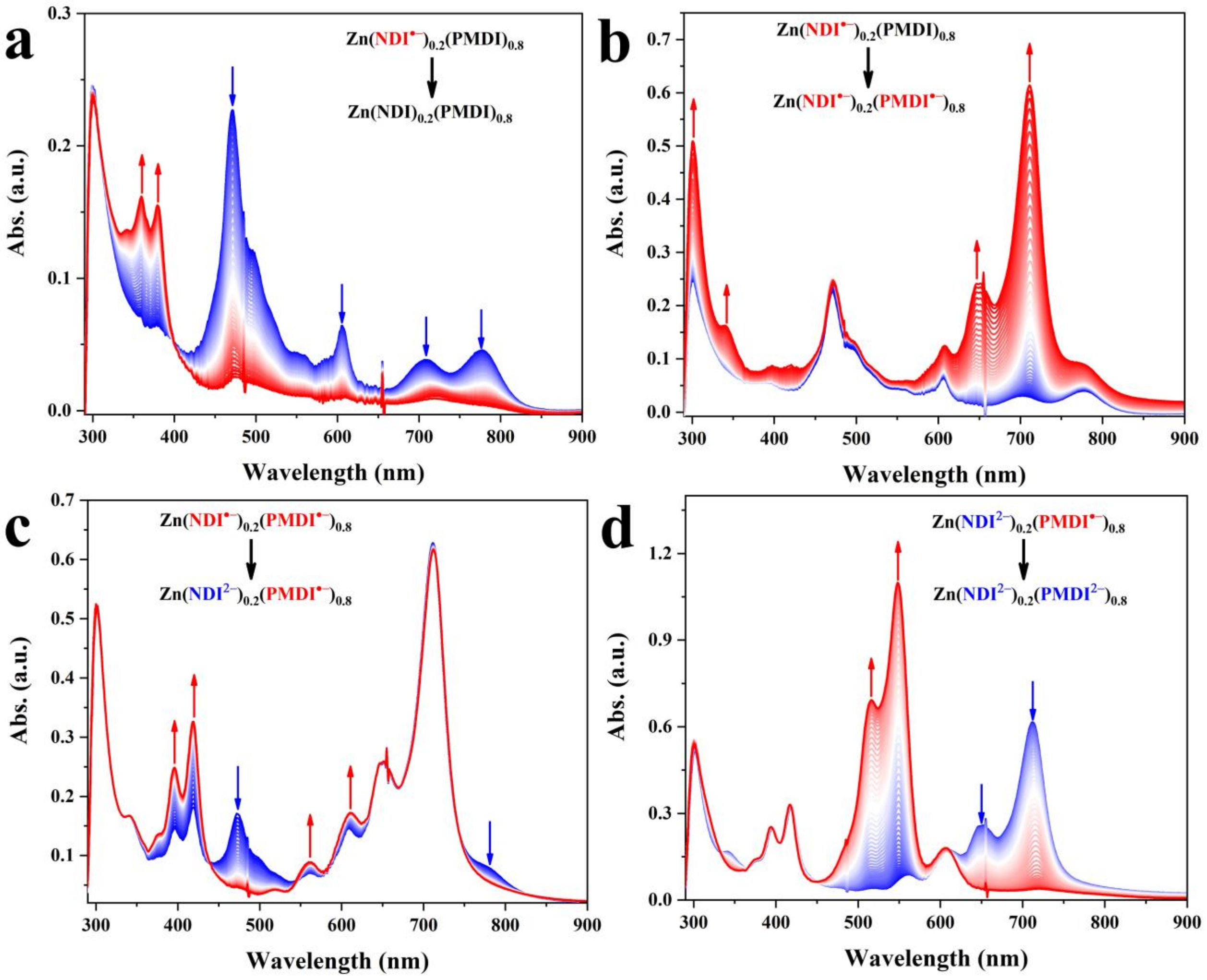

Figure S20. UV-vis spectroelectrochemistry measurements of the Zn(NDI)$_{0.2}$(PMDI)$_{0.8}$ thin film while slowing reducing it with finely modulated applied potential to access the vibrational signature of different redox states, singly reduced mono-radical state Zn(NDI$^{\bullet-}$)$_{0.2}$(PMDI)$_{0.8}$ (a), doubly reduced mixed radical state Zn(NDI$^{\bullet-}$)$_{0.2}$(PMDI$^{\bullet-}$)$_{0.8}$ (b), triply reduced mixed radical-dianion state Zn(NDI^{2-})$_{0.2}$(PMDI$^{\bullet-}$)$_{0.8}$ (c), and quadruply reduced mixed dianion state Zn(NDI^{2-})$_{0.2}$(PMDI^{2-})$_{0.8}$ (d), sorting by the formation of characteristic anion or dianion species in sequence.

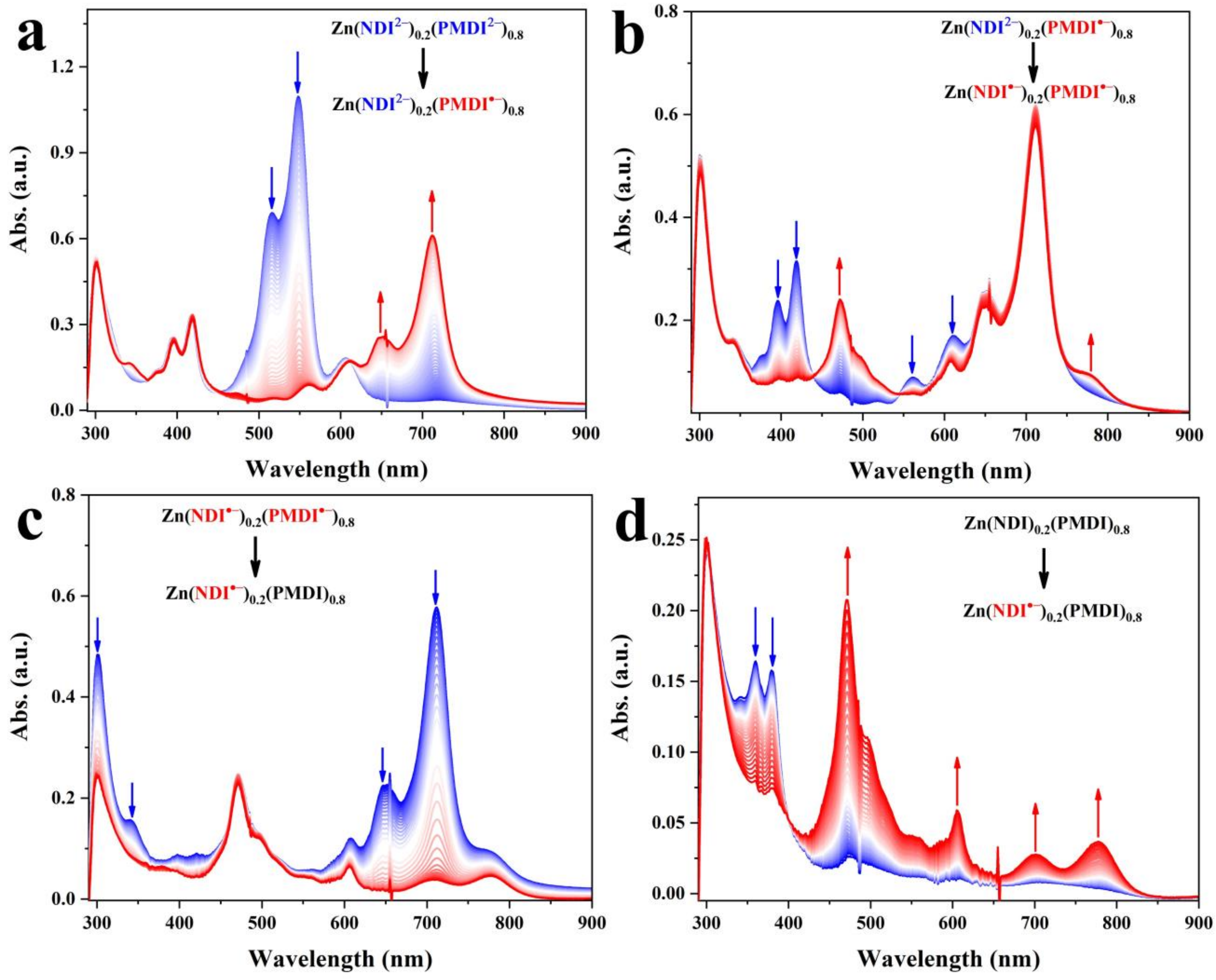

Figure S21. UV-vis spectroelectrochemistry measurements of the $Zn(NDI)_{0.2}(PMDI)_{0.8}$ thin film while slowing re-oxidizing it with finely modulated applied potential to access the vibrational signature of different redox states, from quadruply reduced mixed dianion state $Zn(NDI^{2-})_{0.2}(PMDI^{2-})_{0.8}$ to triply reduced mixed radical-dianion state $Zn(NDI^{2-})_{0.2}(PMDI^{\bullet -})_{0.8}$ (a), then doubly reduced mixed radical state $Zn(NDI^{\bullet -})_{0.2}(PMDI^{\bullet -})_{0.8}$ (b), followed by singly reduced mono-radical state $Zn(NDI^{\bullet -})_{0.2}(PMDI)_{0.8}$ (c), and finally neutral $Zn(NDI)_{0.2}(PMDI)_{0.8}$ (d).

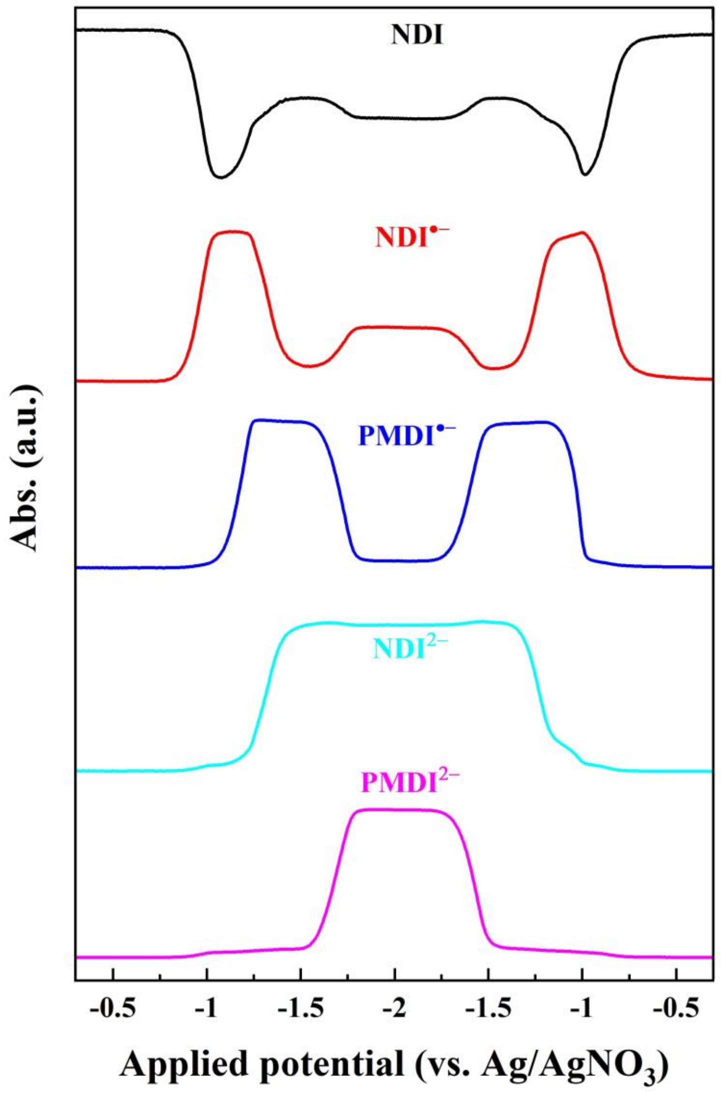

Figure S22. The evolution of the five distinct electronic states as the function of applied potential in the $Zn(NDI)_{0.2}(PMDI)_{0.8}$ thin film. Characteristic absorptions at 360 nm (NDI), 471 nm ($NDI^{•−}$), 713 nm ($PMDI^{•−}$), 418 nm (NDI^{2-}), and 551 nm ($PMDI^{2-}$) were selected to represent those electronic states.

3.5. $Zn(NDI)_{0.8}(PMDI)_{0.2}$ thin film

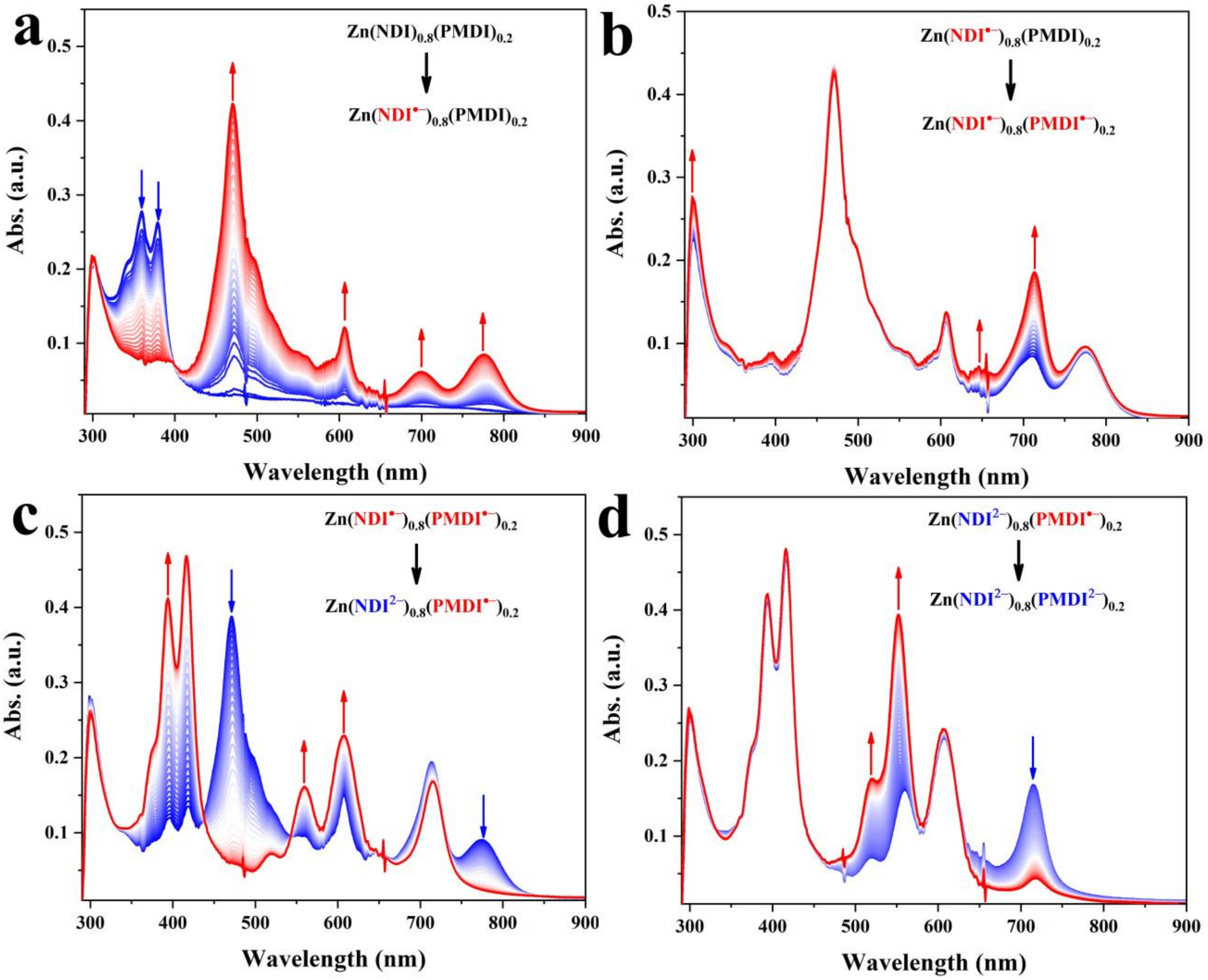

Figure S23. UV-vis spectroelectrochemistry measurements of the $Zn(NDI)_{0.8}(PMDI)_{0.2}$ thin film while slowing reducing it with finely modulated applied potential to access the vibrational signature of different redox states, singly reduced mono-radical state $Zn(NDI^{\bullet-})_{0.8}(PMDI)_{0.2}$ (a), doubly reduced mixed radical state $Zn(NDI^{\bullet-})_{0.8}(PMDI^{\bullet-})_{0.2}$ (b), triply reduced mixed radical-dianion state $Zn(NDI^{2-})_{0.8}(PMDI^{\bullet-})_{0.2}$ (c), and quadruply reduced mixed dianion state $Zn(NDI^{2-})_{0.8}(PMDI^{2-})_{0.2}$ (d), sorting by the formation of characteristic anion or dianion species in sequence.

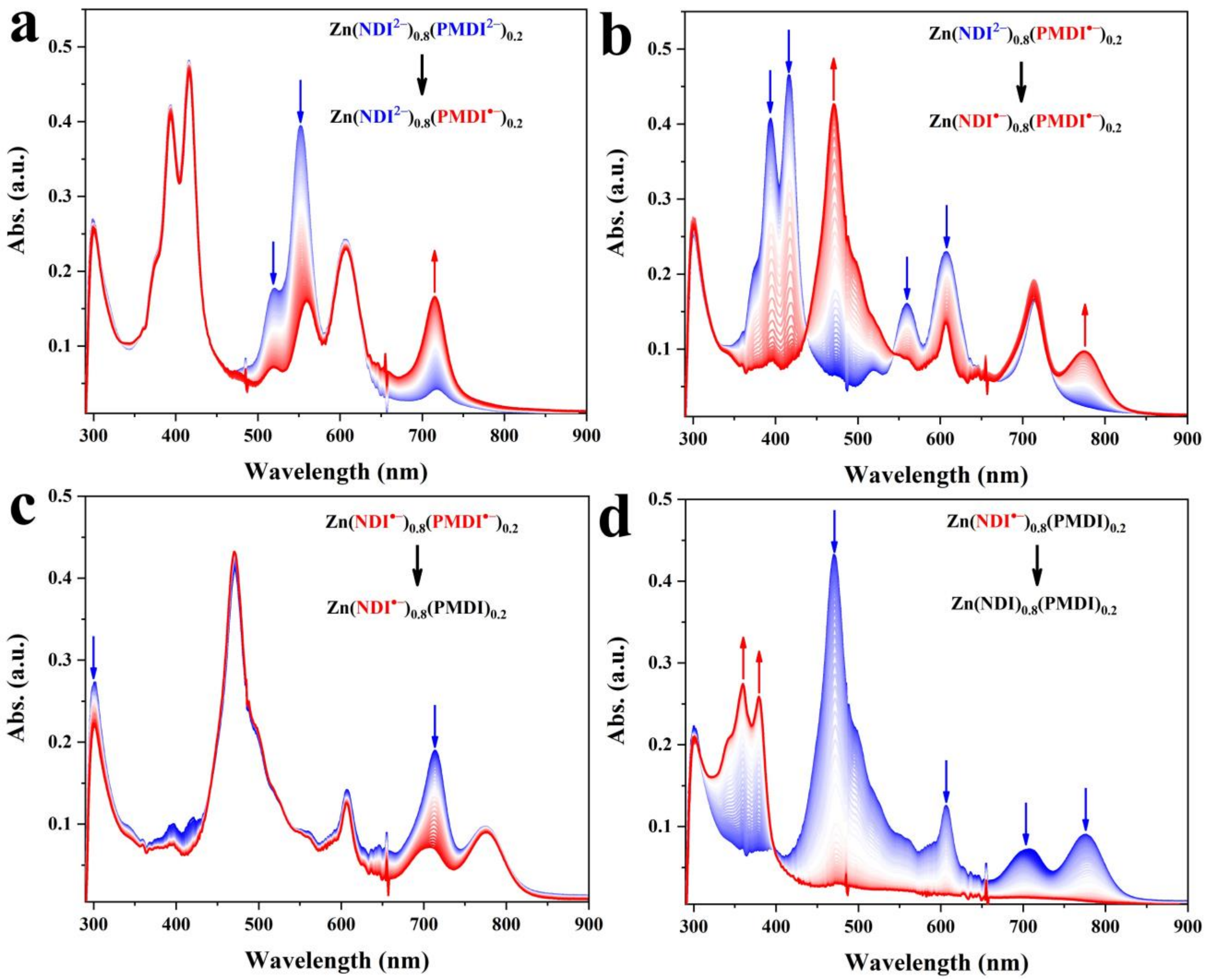

Figure S24. UV-vis spectroelectrochemistry measurements of the Zn(NDI)$_{0.8}$(PMDI)$_{0.2}$ thin film while slowing re-oxidizing it with finely modulated applied potential to access the vibrational signature of different redox states, from quadruply reduced mixed dianion state Zn(NDI^{2-})$_{0.8}$(PMDI^{2-})$_{0.2}$ to triply reduced mixed radical-dianion state Zn(NDI^{2-})$_{0.8}$(PMDI$^{•-}$)$_{0.2}$ (a), then doubly reduced mixed radical state Zn(NDI$^{•-}$)$_{0.8}$(PMDI$^{•-}$)$_{0.2}$ (b), followed by singly reduced mono-radical state Zn(NDI$^{•-}$)$_{0.8}$(PMDI)$_{0.2}$ (c), and finally neutral Zn(NDI)$_{0.8}$(PMDI)$_{0.2}$ (d).

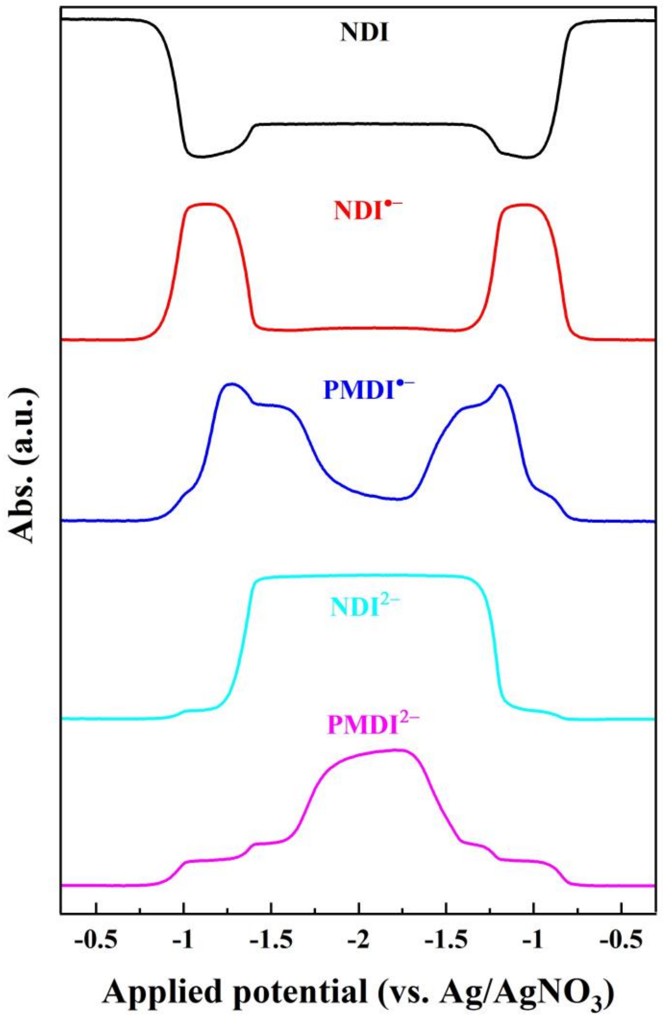

Figure S25. The evolution of the five distinct electronic states as the function of applied potential in the $Zn(NDI)_{0.8}(PMDI)_{0.2}$ thin film. Characteristic absorptions at 360 nm (NDI), 471 nm ($NDI^{\bullet-}$), 713 nm ($PMDI^{\bullet-}$), 418 nm (NDI^{2-}), and 551 nm ($PMDI^{2-}$) were selected to represent those electronic states.

4. Pulsed step-potential spectrochronoamperometry studies

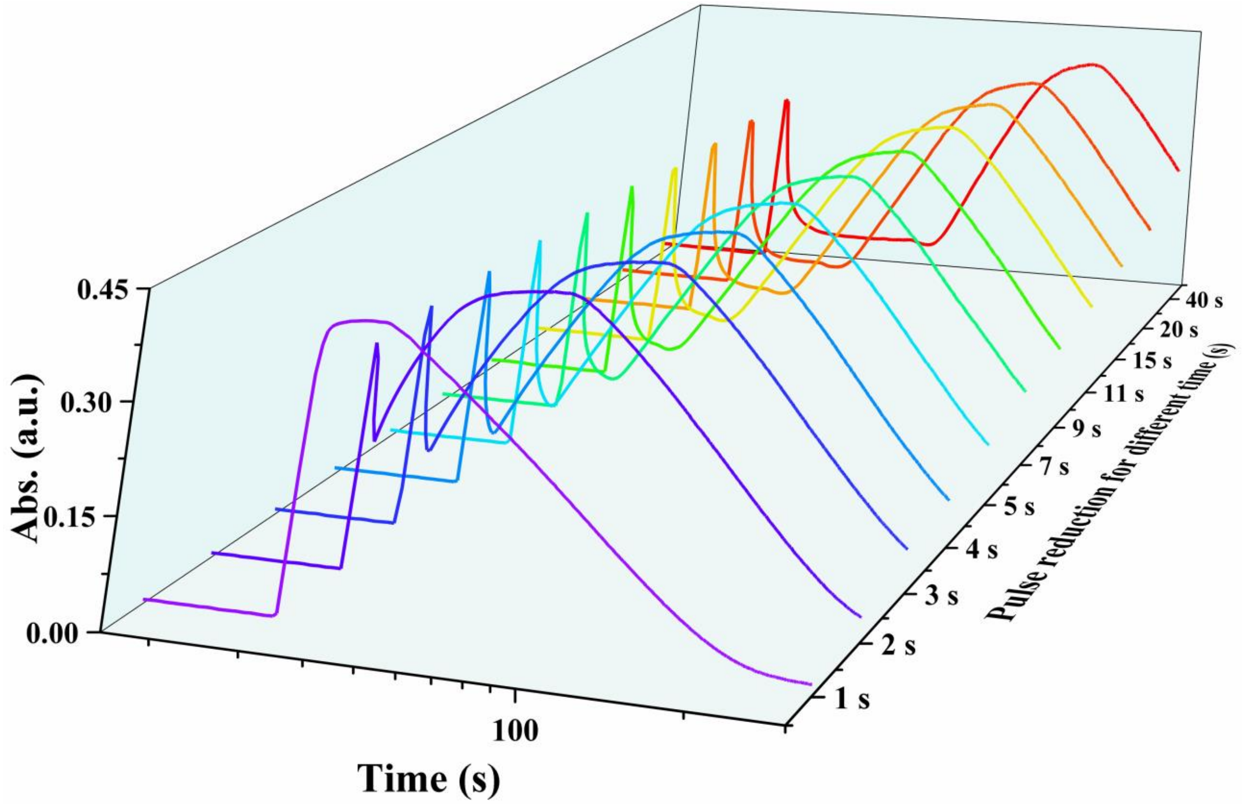

Figure S26. Pulsed step-potential spectrochronoamperometry monitoring the evolution of NDI$^{\bullet-}$ (471 nm) after stepping the potential from -0.2 V to -1.9 V vs Ag/AgNO$_3$ followed by open circuit operation, the pulse was prolonged from 1 to 40 s.

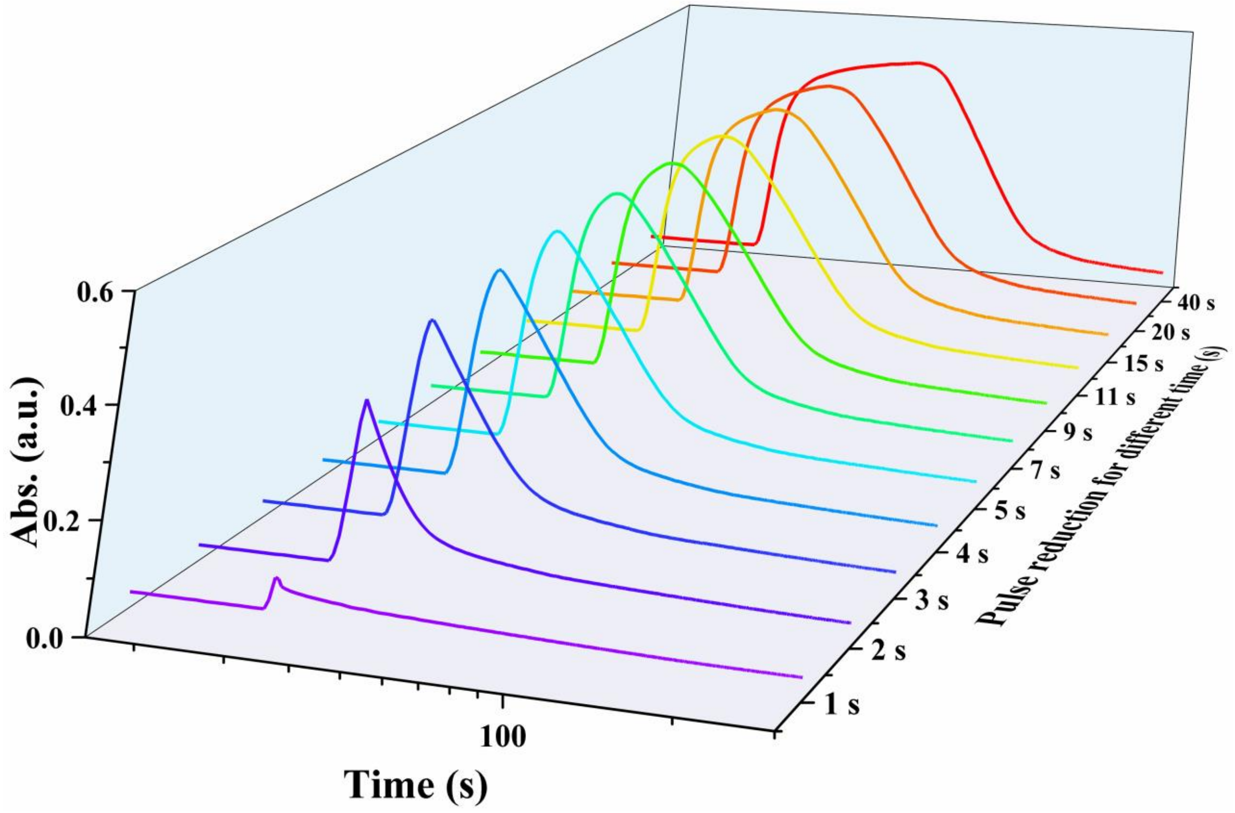

Figure S27. Pulsed step-potential spectrochronoamperometry monitoring the evolution of NDI^{2-} (418 nm) after stepping the potential from -0.2 V to -1.9 V vs $Ag/AgNO_3$ followed by open circuit operation, the pulse was prolonged from 1 to 40 s.

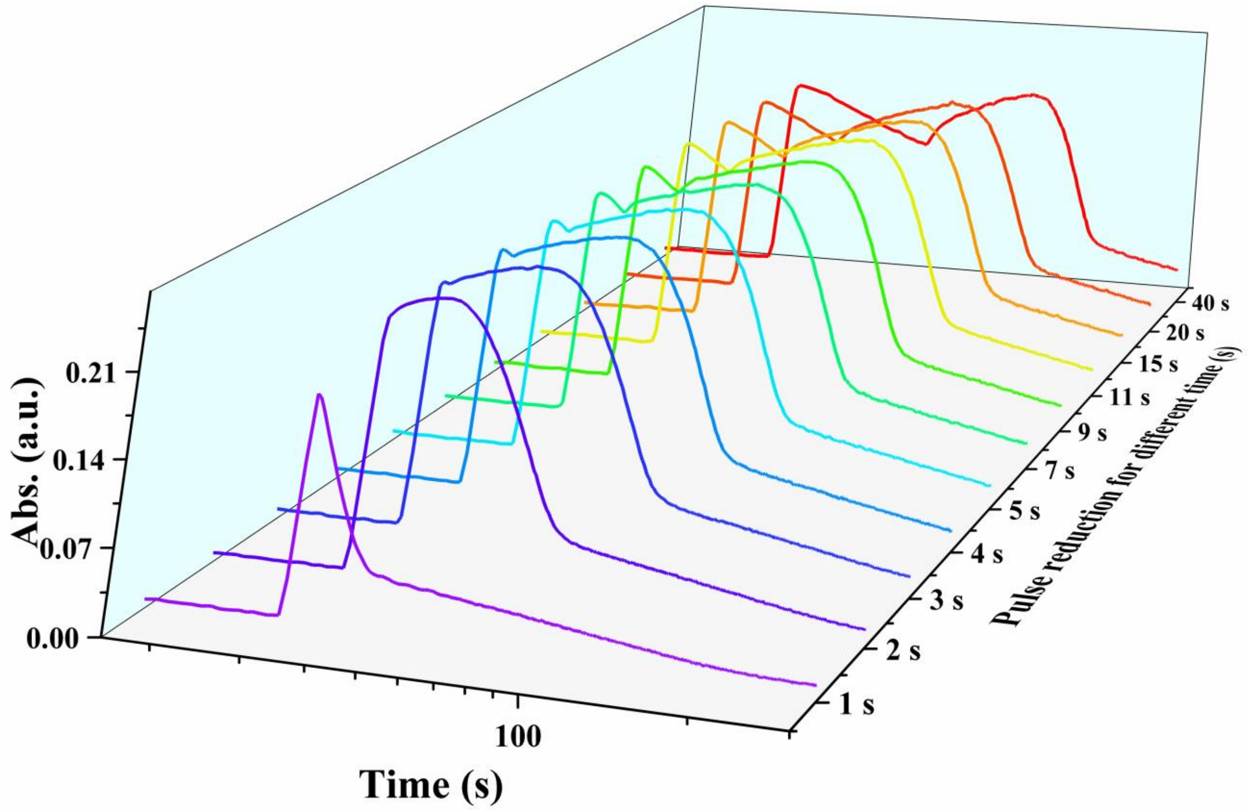

Figure S28. Pulsed step-potential spectrochronoamperometry monitoring the evolution of PMDI$^{\bullet-}$ (713 nm) after stepping the potential from -0.2 V to -1.9 V vs Ag/AgNO$_3$ followed by open circuit operation, the pulse was prolonged from 1 to 40 s.

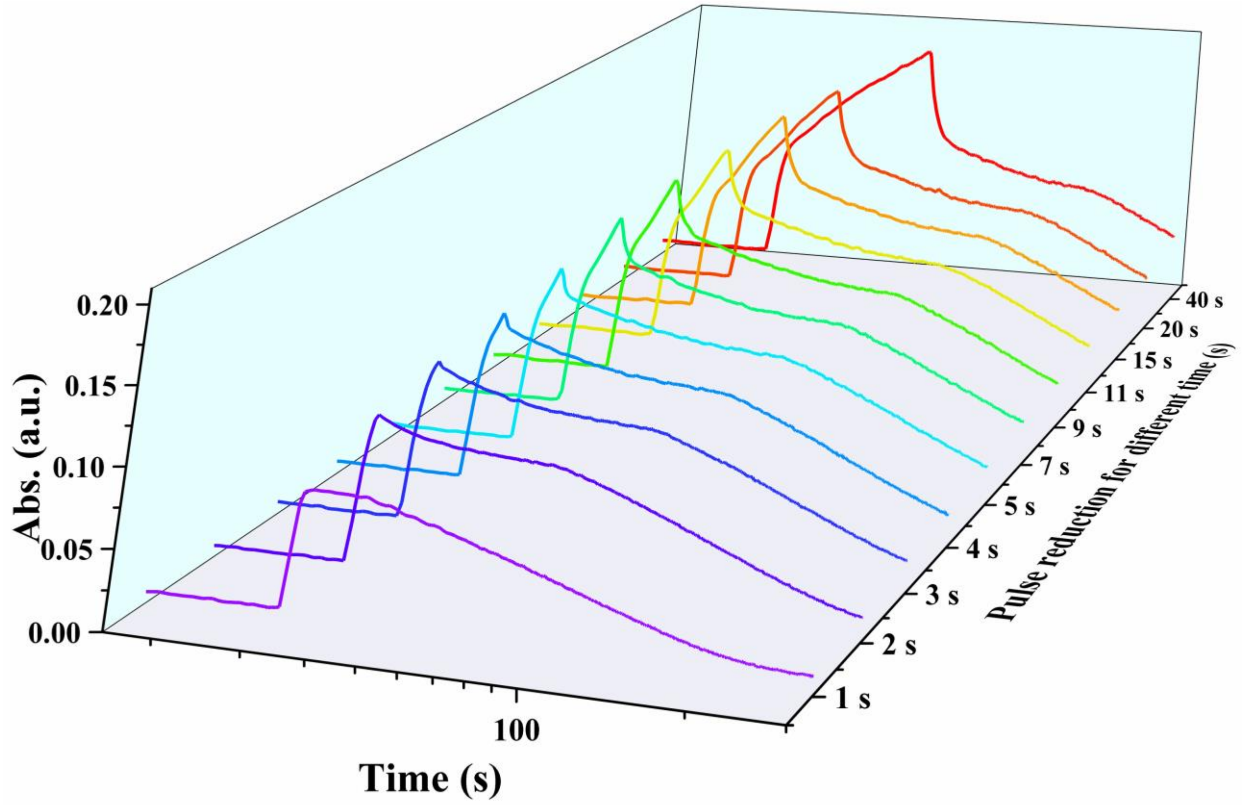

Figure S29. Pulsed step-potential spectrochronoamperometry monitoring the evolution of $PMDI^{2-}$ (551 nm) after stepping the potential from -0.2 V to -1.9 V vs $Ag/AgNO_3$ followed by open circuit operation, the pulse was prolonged from 1 to 40 s.

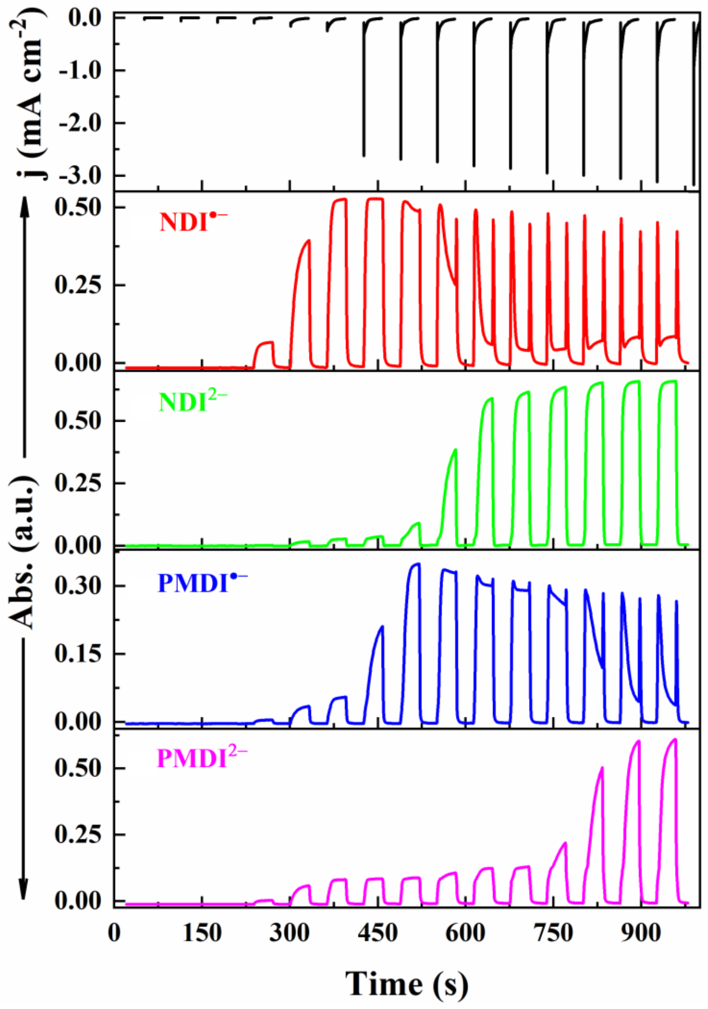

Figure S30. Pulsed (30 s) cathodic step-potential spectrochronoamperometry monitoring the evolution of $NDI^{\bullet-}$ (471 nm), NDI^{2-} (418 nm), $PMDI^{\bullet-}$ (713 nm), and $PMDI^{2-}$ (551 nm) after stepping the potential from 0.0 V vs Ag/$AgNO_3$ to more and more reducing potentials (-0.2, -0.4, -0.6, -0.8, -0.9, -1, -1.1, -1.2, -1.3, -1.4, -1.5, -1.6, -1.7, -1.8, -1.9, -2 V vs Ag/$AgNO_3$). After each step-potential reduction, the film was re-oxidized back to neutral state by applying 0.0 V vs Ag/$AgNO_3$ for 30 s.

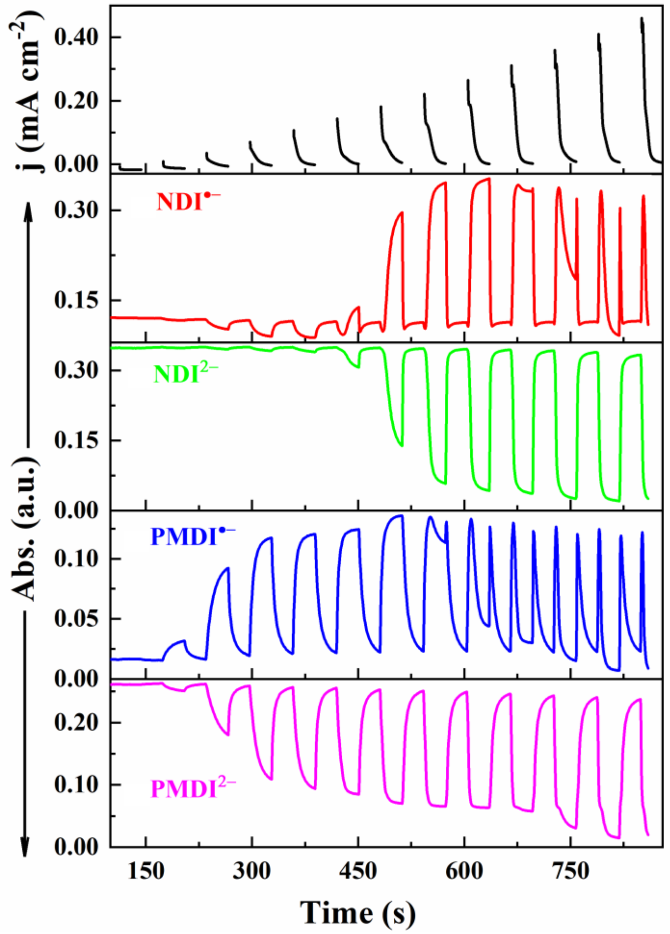

Figure S31. Pulsed (30 s) anodic step-potential spectrochronoamperometry monitoring the evolution of NDI$^{•-}$ (471 nm), NDI^{2-} (418 nm), PMDI$^{•-}$ (713 nm), and PMDI^{2-} (551 nm) after stepping the potential from -2 V vs Ag/AgNO$_3$ to more and more oxidizing potentials (-2, -1.9, -1.8, -1.7, -1.6, -1.5, -1.4, -1.3, -1.2, -1.1, -1, -0.9, -0.8, -0.7 V vs Ag/AgNO$_3$). After each step-potential reduction, the film was re-oxidized back to neutral state by applying -2 V vs Ag/AgNO$_3$ for 30 s.

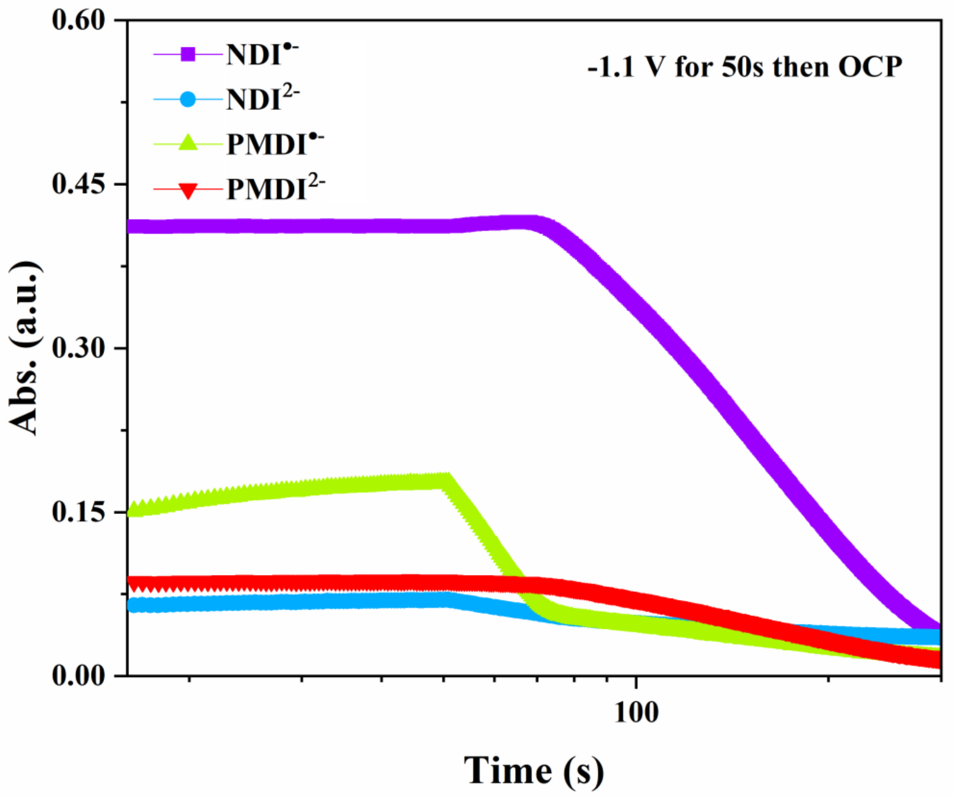

Figure S32. Spectrochronoamperometry monitoring the evolution of $NDI^{•-}$ (471 nm), NDI^{2-} (418 nm), $PMDI^{•-}$ (713 nm), and $PMDI^{2-}$ (551 nm) after holding the potential at -1.1 V vs $Ag/AgNO_3$ for 50 s then followed by open circuit operation to let the system to relax. In the presence of trace amount of atmospheric oxygen molecules, the reduced species will be gradually re-oxidized to neutral state.

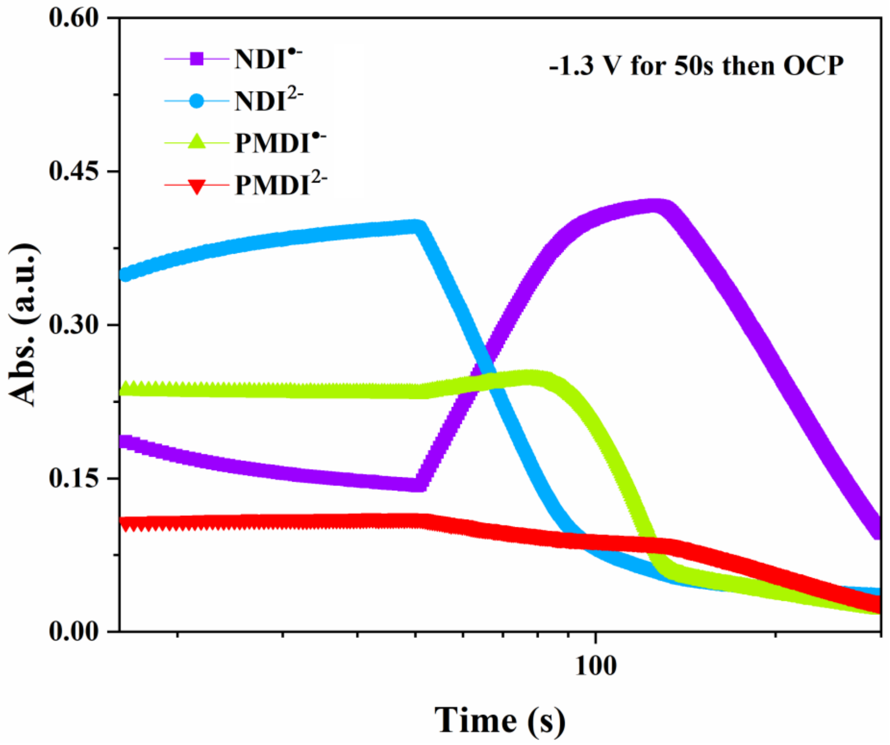

Figure S33. Spectrochronoamperometry monitoring the evolution of $NDI^{•-}$ (471 nm), NDI^{2-} (418 nm), $PMDI^{•-}$ (713 nm), and $PMDI^{2-}$ (551 nm) after holding the potential at -1.3 V vs $Ag/AgNO_3$ for 50 s then followed by open circuit operation to let the system to relax. In the presence of trace amount of atmospheric oxygen molecules, the reduced species will be gradually re-oxidized to neutral state.

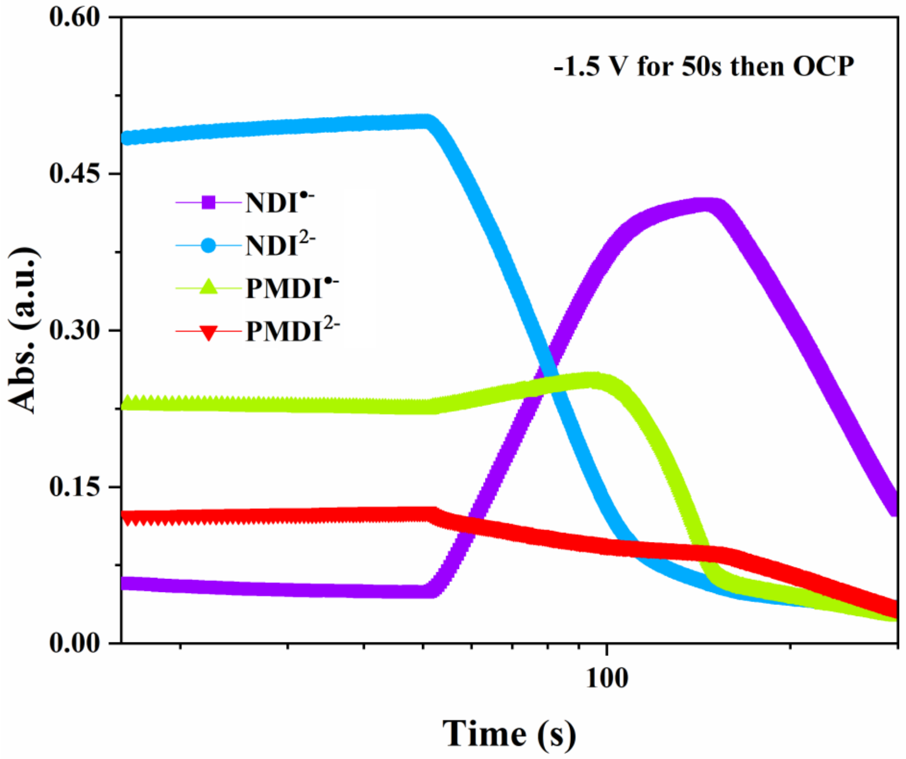

Figure S34. Spectrochronoamperometry monitoring the evolution of NDI•− (471 nm), NDI2- (418 nm), PMDI•− (713 nm), and PMDI2- (551 nm) after holding the potential at -1.5 V vs Ag/AgNO$_3$ for 50 s then followed by open circuit operation to let the system to relax. In the presence of trace amount of atmospheric oxygen molecules, the reduced species will be gradually re-oxidized to neutral state.

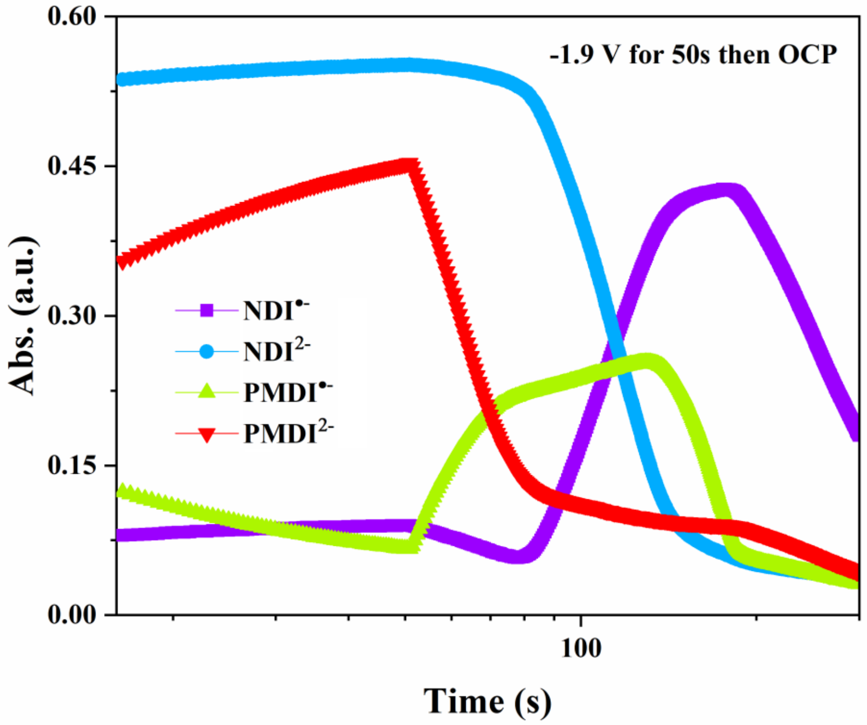

Figure S35. Spectrochronoamperometry monitoring the evolution of NDI•− (471 nm), NDI^{2-} (418 nm), PMDI•− (713 nm), and $PMDI^{2-}$ (551 nm) after holding the potential at -1.9 V vs $Ag/AgNO_3$ for 50 s then followed by open circuit operation to let the system to relax. In the presence of trace amount of atmospheric oxygen molecules, the reduced species will be gradually re-oxidized to neutral state.

5. Steady-state redox conductivity studies

5.1. Equivalent circuits

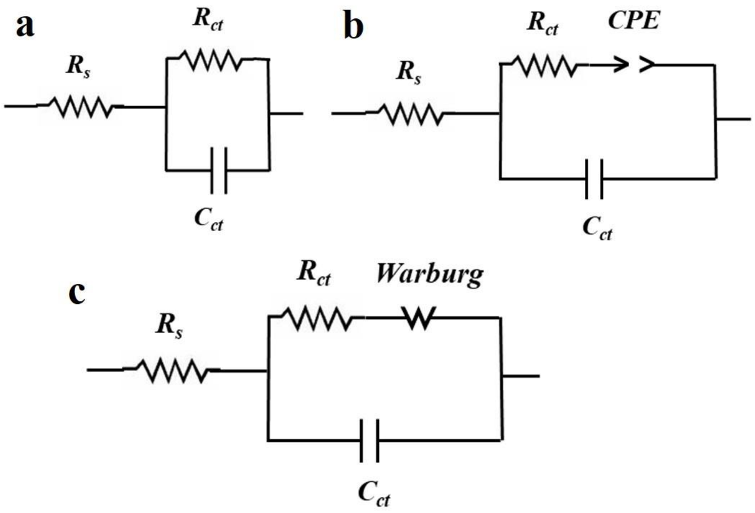

Figure S36 Equivalent circuits (EC) for simulation of the experimental impedance date. (a) Simplified RC circuit, where R_{ct} and C_{ct} stand for resistance and capacitance related to the inter-site cation coupled electron hopping. (b) Modified RC circuit by adding the serial a constant phase element (CPE) to the electronic resistance component, where CPE is primarily defined by its phase, n ($0 \leq n \leq 1$), when n equals 1, 0.5, or 0, the CPE represent an ideal capacitor, a semi-infinite diffusional Warburg element, and an ideal resistor, respectively. (c) Modified RC circuit by adding the serial Warburg element to the electronic resistance component. In all circuits, *Rs* stands for the resistance related to electrolyte.

5.2. Zn(NDI) thin film

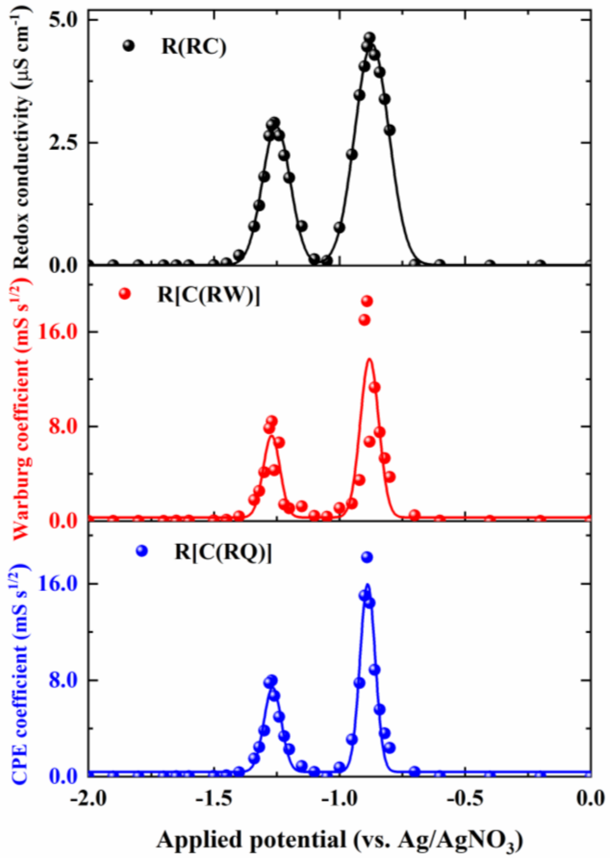

Figure S37. Evolution of redox conductivity (upper panel, simulated from **Fig. S36a**), Warburg coefficient (middle panel, simulated from **Fig. S36c**) and CPE coefficient (lower panel, simulated from **Fig. S36b**) as the function of electrochemical potential (of the thin-film) measured in 0.1 M KPF_6 DMF electrolyte, which is determined by the redox state of the Zn(NDI) thin film. Note that the unit of the Y-axes for lower panel depends upon the phase, *n*; to facilitate comparison, *n* was assumed to be 0.5 here. Gaussian fit was performed for the NDI/$NDI^{\bullet-}$ based and $NDI^{\bullet-}$/NDI^{2-} based bell-shaped redox conductivities.

5.3. $Zn(NDI)_{0.2}(PMDI)_{0.8}$ thin film

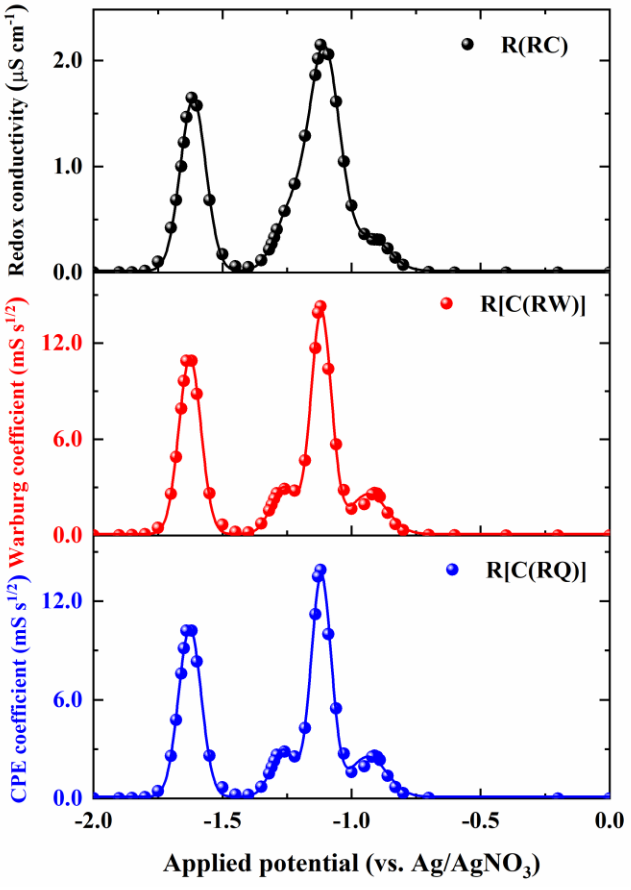

Figure S38. Evolution of redox conductivity (upper panel, simulated from **Fig. S36a**), Warburg coefficient (middle panel, simulated from **Fig. S36c**) and CPE coefficient (lower panel, simulated from **Fig. S36b**) as the function of electrochemical potential (of the thin-film) measured in 0.1 M KPF_6 DMF electrolyte, which is determined by the redox state of the $Zn(NDI)_{0.2}(PMDI)_{0.8}$ thin film. Note that the unit of the Y-axes for lower panel depends upon the phase, *n*; to facilitate comparison, *n* was assumed to be 0.5 here. Gaussian fit was performed for the NDI/$NDI^{•-}$ based, PMDI/$PMDI^{•-}$ based, $NDI^{•-}$/NDI^{2-} based and $PMDI^{•-}$/$PMDI^{2-}$ based bell-shaped redox conductivities.

5.4. $Zn(NDI)_{0.5}(PMDI)_{0.5}$ thin film

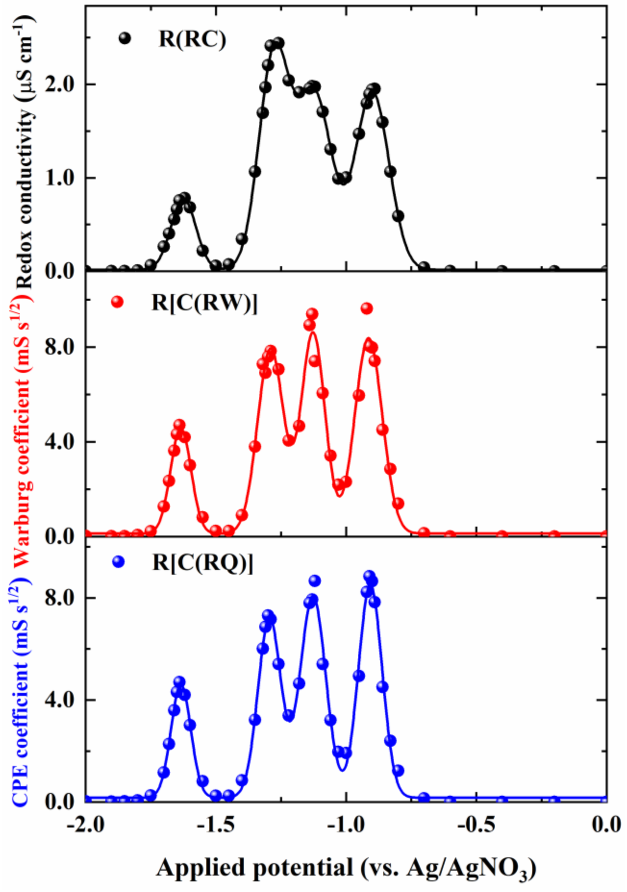

Figure S39. Evolution of redox conductivity (upper panel, simulated from **Fig. S36a**), Warburg coefficient (middle panel, simulated from **Fig. S36c**) and CPE coefficient (lower panel, simulated from **Fig. S36b**) as the function of electrochemical potential (of the thin-film) measured in 0.1 M KPF_6 DMF electrolyte, which is determined by the redox state of the $Zn(NDI)_{0.5}(PMDI)_{0.5}$ thin film. Note that the unit of the Y-axes for lower panel depends upon the phase, *n*; to facilitate comparison, *n* was assumed to be 0.5 here. Gaussian fit was performed for the NDI/$NDI^{\bullet-}$ based, PMDI/$PMDI^{\bullet-}$ based, $NDI^{\bullet-}$/NDI^{2-} based and $PMDI^{\bullet-}$/$PMDI^{2-}$ based bell-shaped redox conductivities.

5.5. $Zn(NDI)_{0.8}(PMDI)_{0.2}$ thin film

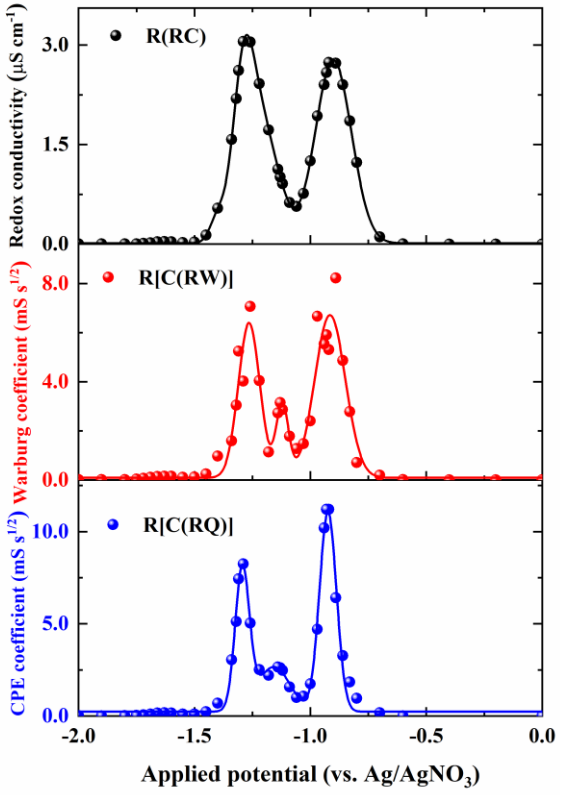

Figure S40. Evolution of redox conductivity (upper panel, simulated from **Fig. S36a**), Warburg coefficient (middle panel, simulated from **Fig. S36c**) and CPE coefficient (lower panel, simulated from **Fig. S36b**) as the function of electrochemical potential (of the thin-film) measured in 0.1 M KPF_6 DMF electrolyte, which is determined by the redox state of the $Zn(NDI)_{0.8}(PMDI)_{0.2}$ thin film. Note that the unit of the Y-axes for lower panel depends upon the phase, *n*; to facilitate comparison, *n* was assumed to be 0.5 here. Gaussian fit was performed for the NDI/NDI$^{•-}$ based, PMDI/PMDI$^{•-}$ based, NDI$^{•-}$/NDI^{2-} based and PMDI$^{•-}$/PMDI^{2-} based bell-shaped redox conductivities.

5.6. Zn(PMDI) thin film

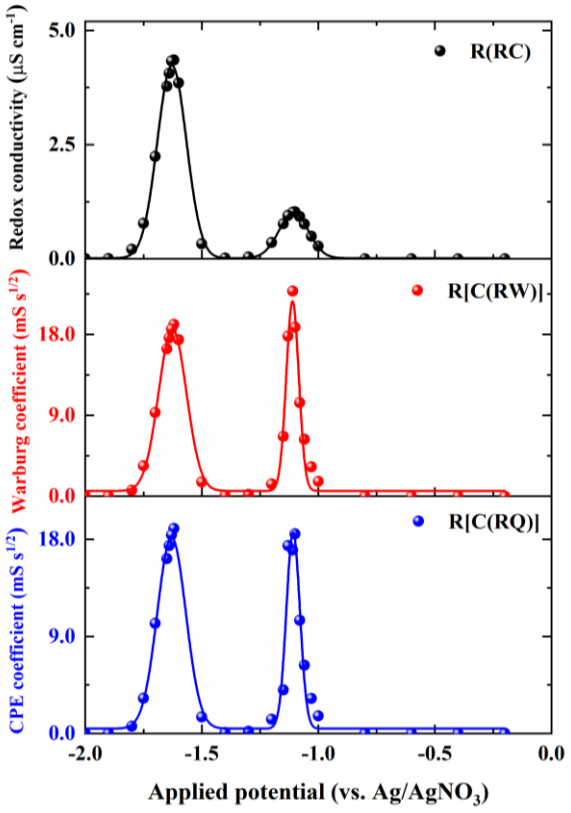

Figure S41. Evolution of redox conductivity (upper panel, simulated from **Fig. S36a**), Warburg coefficient (middle panel, simulated from **Fig. S36c**) and CPE coefficient (lower panel, simulated from **Fig. S36b**) as the function of electrochemical potential (of the thin-film) measured in 0.1 M KPF_6 DMF electrolyte, which is determined by the redox state of the Zn(PMDI) thin film. Note that the unit of the Y-axes for lower panel depends upon the phase, *n*; to facilitate comparison, *n* was assumed to be 0.5 here. Gaussian fit was performed for the PMDI/$PMDI^{\bullet-}$ based and $PMDI^{\bullet-}$/$PMDI^{2-}$ based bell-shaped redox conductivities.
